# Collagen IV^α345^ dysfunction in glomerular basement membrane diseases. II. Crystal structure of the α345 hexamer

**DOI:** 10.1016/j.jbc.2021.100591

**Published:** 2021-03-26

**Authors:** Sergei P. Boudko, Ryan Bauer, Sergei V. Chetyrkin, Sergey Ivanov, Jarrod Smith, Paul A. Voziyan, Billy G. Hudson

**Affiliations:** 1 Department of Medicine, Division of Nephrology and Hypertension, Vanderbilt University Medical Center, Nashville, Tennessee, USA; 2 Center for Matrix Biology, Vanderbilt University Medical Center, Nashville, Tennessee, USA; 3 Department of Biochemistry, Center for Structural Biology, Vanderbilt University, Nashville, Tennessee, USA; 4 Aspirnaut, Vanderbilt University Medical Center, Nashville, Tennessee, USA; 5 Department of Pathology, Microbiology and Immunology, Vanderbilt University Medical Center, Nashville, Tennessee, USA; 6 Department of Cell and Developmental Biology, Vanderbilt University, Nashville, Tennessee, USA; 7 Vanderbilt Institute of Chemical Biology, Vanderbilt University, Nashville, Tennessee, USA; 8 Vanderbilt-Ingram Cancer Center, Vanderbilt University, Nashville, Tennessee, USA

**Keywords:** extracellular matrix, collagen, X-ray crystallography, atomic force microscopy (AFM), genetic disease, AS, Alport syndrome, DN, diabetic nephropathy, GBM, glomerular basement membrane, GP, Goodpasture’s disease, LCL, loop-crevice-loop

## Abstract

Our recent work identified a genetic variant of the α345 hexamer of the collagen IV scaffold that is present in patients with glomerular basement membrane diseases, Goodpasture’s disease (GP) and Alport syndrome (AS), and phenocopies of AS in knock-in mice. To understand the context of this “Zurich” variant, an 8-amino acid appendage, we developed a construct of the WT α345 hexamer using the single-chain NC1 trimer technology, which allowed us to solve a crystal structure of this key connection module. The α345 hexamer structure revealed a ring of 12 chloride ions at the trimer–trimer interface, analogous to the collagen α121 hexamer, and the location of the 170 AS variants. The hexamer surface is marked by multiple pores and crevices that are potentially accessible to small molecules. Loop-crevice-loop features constitute bioactive sites, where pathogenic pathways converge that are linked to AS and GP, and, potentially, diabetic nephropathy. In Pedchenko *et al*., we demonstrate that these sites exhibit conformational plasticity, a dynamic property underlying assembly of bioactive sites and hexamer dysfunction. The α345 hexamer structure is a platform to decipher how variants cause AS and how hypoepitopes can be triggered, causing GP. Furthermore, the bioactive sites, along with the pores and crevices on the hexamer surface, are prospective targets for therapeutic interventions.

Prominent diseases of the glomerular basement membrane (GBM), a specialized form of the extracellular matrix, are diabetic nephropathy (DN), Alport syndrome (AS), and Goodpasture’s disease (GP). The morphological abnormalities in the GBM involve structural alterations in collagen IV^α345^ scaffold, the major GBM component (1, 2, 3). The mechanisms whereby collagen IV enables normal GBM function or causes GBM abnormalities and dysfunction in disease are unknown.

In Pokidysheva *et al*. (4), we found that the α345 hexamer, a key connection module within the collagen IV^α345^ scaffold, is a focal point of bioactivity within the GBM, based on investigation of the Zurich variant that caused AS. To understand how variants, including the Z-variant, in AS cause renal dysfunction, knowledge of the 3D structure of the α345 hexamer is critical. Moreover, this knowledge is also critical to understanding renal dysfunction in GP and DN and development of therapies. Therefore, we solved a crystal structure of the α345NC1 hexamer, a goal that has been pursued by scientists for several decades. The crystal structure revealed features critical for GBM function and in pathogenesis of AS and GP, and, potentially, DN, thus providing a framework for the development of therapies.

## Results

After decades of attempts to isolate and crystallize the α345 hexamer, we developed a recombinant single-chain NC1 trimer technology (5) and used it to define the arrangement of chains and solve the crystal structure of the recombinant α345 hexamer.

### Composition and arrangement of chains within the α345 hexamer

**Figure 1:**
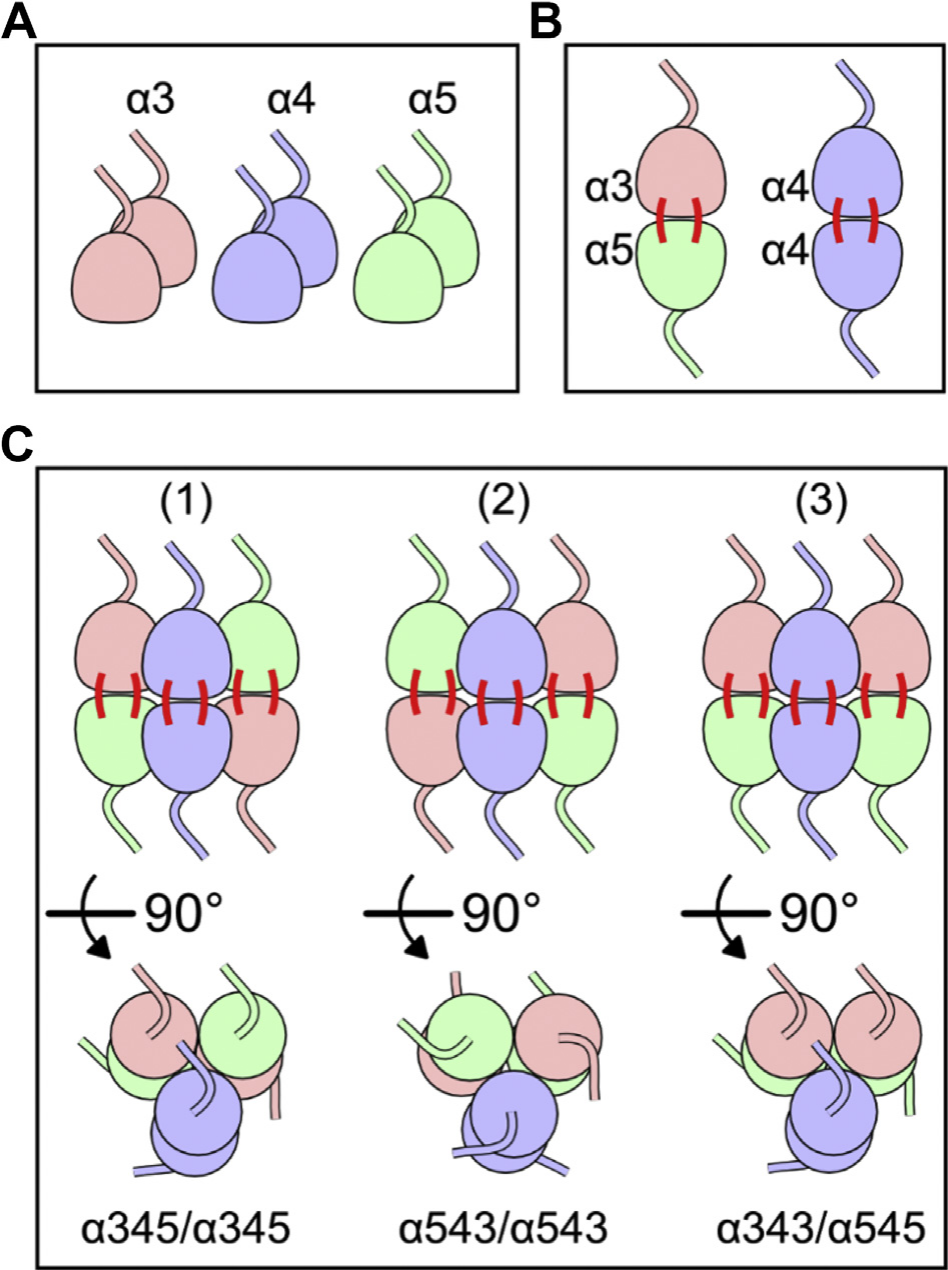
**Possible chain orientations within the α345 hexamer.** We previously found that the GBM NC1^α345^ hexamer contains two copies of the α3 (*light red*), α4 (*light blue*), and α5 (*light green*) NC1 monomers (*A*) and sulfilimine crosslinking of opposite chains forming two types of dimers, α3-α5 and α4-α4 (*B*) (7). Taking into account the composition and sulfilimine crosslinking pattern, three chain orientations are possible within the NC1^α345^ hexamer (*C*). GBM, glomerular basement membrane.

The collagen IV^α345^ scaffold, composed of the α3, α4, and α5 chains, is a major constituent of the GBM. The α345 hexamer can be extracted from the GBM using collagenase treatment (6). We previously determined the equimolar composition of α3, α4, and α5 chains in the α345 hexamers isolated from the GBM (7) (Fig. 1*A*). In the same study, we also found the presence of α3-α5 and α4-α4 covalently linked dimers (7), where chain monomers from opposite trimers were connected by sulfilimine bonds (8) (Fig. 1*B*). However, the exact chain arrangement within the hexamer is unknown. Taking into account our previous findings, there are only three possible chain arrangements in the α345 hexamers: (1) α345-to-α345, (2) α543-to-α543, and (3) α343-to-α545 (Fig. 1*C*).

**Figure 2:**
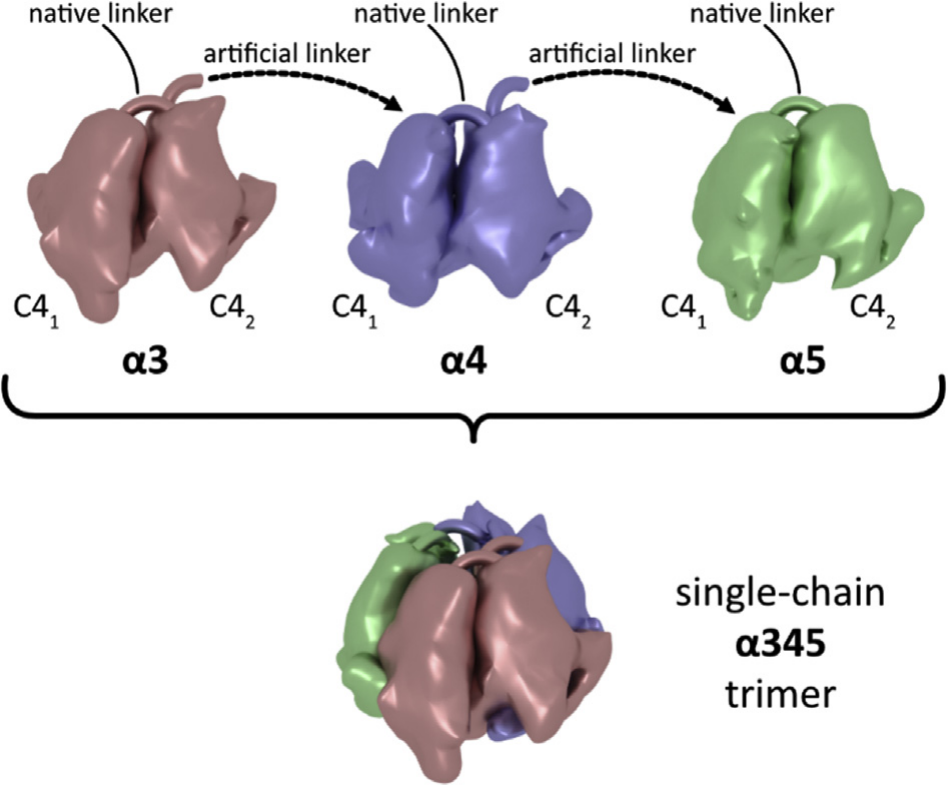
**Schematic presentation of the single-chain technology used for generating the α345 NC1 trimer.** A single polypeptide combines NC1 monomers of α3, α4, and α5 chains *via* artificial linkers analogous to native linkers between C4 subdomains within each monomer. Short linkers restrict orientation of monomers in a way that the C4_2_ subdomain of the α3 chain will interface with the C4_1_ subdomain of the α4 chain and the C4_2_ subdomain of the α4 chain will interface with the C4_1_ subdomain of the α5 chain. Finally, the C4_2_ subdomain of the α5 chain will interface with the C4_1_ subdomain of the α3 chain in the folded single-chain α345 NC1 trimer. The same strategy was applied to generate α543, α343, and α545 forms of the single-chain NC1 trimer.

We tested these possibilities experimentally using the single-chain NC1 trimer technology (5) developed and verified for the α121 hexamer. This technology allows to define composition and orientation of the chains in the trimer (Fig. 2). The α345, α543, α343, and α545 NC1 single-chain NC1 trimers carrying the signal peptide for secretion (Fig. S1) were transiently expressed in expiCHO cells. Total cell lysates and media were analyzed for the presence of protein of interest using Western blotting (Fig. S2). Only α345 and α545 constructs were detected in the media, whereas α543 and α343 were exclusively trapped within the cells, indicating misfolding problem. Although α545 was partially secreted to the medium, the required partner, α343, was trapped within the cells. Coexpression of α343 and α545 did not rescue secretion of α343. Collectively, the single-chain α345 NC1 trimer represents native composition and orientation of chains. This is also supported by previous studies where association of individual α4 or α5 NC1 monomers with the α3 chain was selectively blocked by the mAbs (7).

### Crystal structure of the α345 hexamer

**Figure 3:**
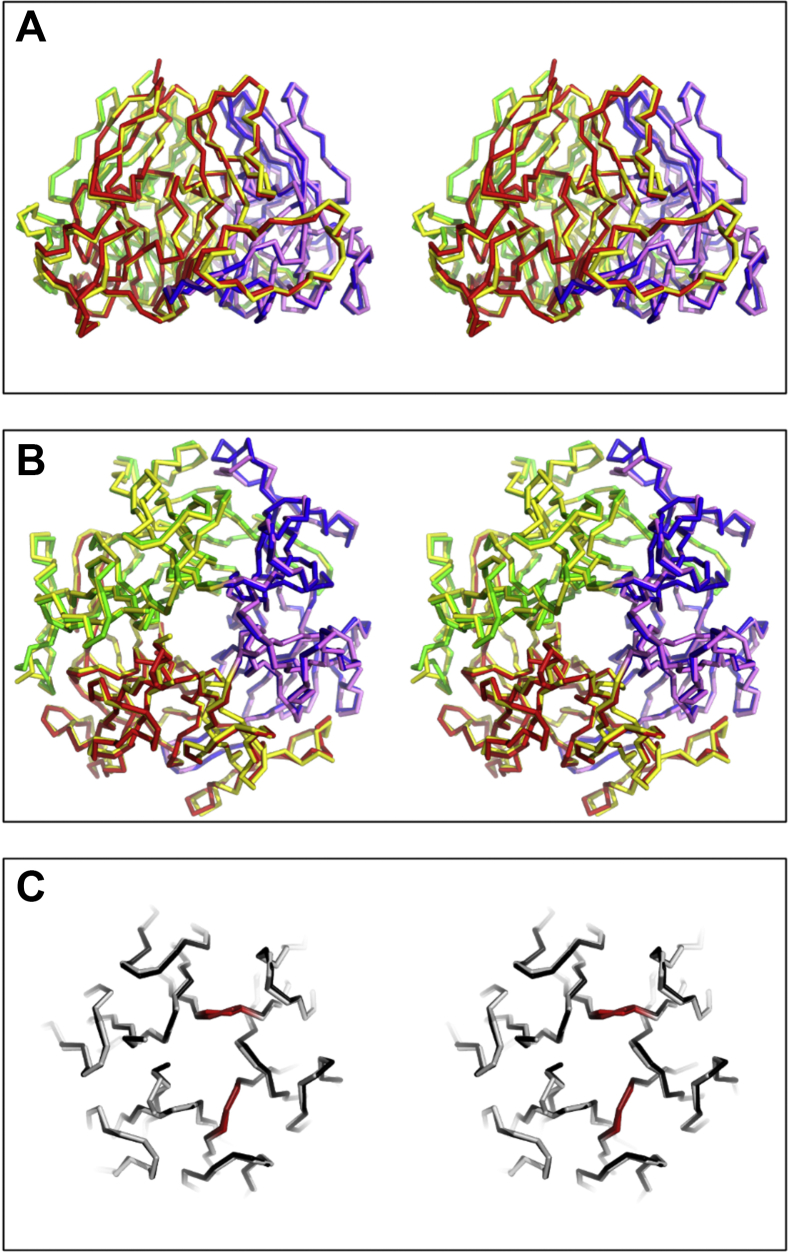
**Crystal structure comparison of the single-chain α345 (this study) and α121 NC1 trimers (PDB code: 6mpx)** (5). The side view (*A*) and top view (*B*) of the superposition of α345 and α121 shown as stereopairs with the wireframe backbone. The α345 NC1 trimer coloring is *red* for α3, *blue* for α4, and *green* for α5. The α121 NC1 trimer coloring is *yellow* for α1 and *violet* for α5. *C*, the top slab of the top view showing the superposition of α345 (*white*) and α121 (*black*) shown as a stereopair with a wireframe backbone. Artificial linkers between chains are shown in *red*.

The single-chain α345 NC1 trimer produced recombinantly in stably transfected HEK293 cells was purified to homogeneity (Fig. S3) and crystallized in the presence of sodium chloride. The crystal structure was solved at the 1.76 Å resolution with a single polypeptide chain per asymmetric unit (Table S1). It has the designed orientation of α chains in the order of α3-to-α4-to-α5 (Figs. 2 and 3). The atomic structure is homologous to the crystal structure of the α121 NC1 domain (5) (Fig. 3), which is also homologous to all reported crystal structures of tissue extracted from the human and bovine α121 NC1 domain (9, 10, 11). Least-square superpositions of whole α345 and α121 trimers and individual chains revealed no significant variations between corresponding C_α_ atoms (overall r.m.s.d. 0.67 Å) (Table S2). Remarkably, α4 chain has the highest r.m.s.d. value of 2.03 Å when superposed with the α2 chain, although still in the range for highly homologous proteins (12). Most of the structural difference is due to presence of two extra residues, which is unique for α4 chain, within the bottom loop, making a contact with the adjacent α5 chain (Fig. 3). Superposition of α3 and α5 chains with corresponding α1 chains has only 0.55 Å and 0.54 Å r.m.s.d. values, which are typical even for identical proteins (12). Structures of α3 and α5 chains in the α345 NC1 trimer are also identical to the crystal structures observed in α3 and α5 homotrimers/homohexamers, r.m.s.d. of 0.55 to 0.60 Å for α3 and 0.58 Å for α5 (Table S3). Nevertheless, NC1 domains demonstrate sufficient plasticity by forming α2 homotetramer/homo-octamer and α4 homohexamer/homododecamer upon crystallization (13), although for the price of ∼20% and ∼10% of their inner sequences being unstructured. Superposition of the α4 structure of the α345 trimer with the structured part of α4 in the artefactual α4 homohexamer/homododecamer has r.m.s.d. values in the range 4.50 to 4.74 Å, pointing to significant variations even for the structured part (Table S3).

High homology and identity of the structures of α3, α4, and α5 chains in the α345 NC1 trimer with the α1 and α2 chains in the NC1 trimer and with α3 and α5 chains in homotrimers/homohexamers also verifies the correct design of the artificial linkers connecting α3-to-α5 and α4-to-α5. All C4 subdomain linkers, native and artificially introduced, are well structured (Fig. 3*C*), related by a pseudohexagonal symmetry and have comparable atomic displacement factors (Fig. S8 in Supporting Section 3), which further verifies the design of artificial linkers.

Analysis of the crystal structure of the single-chain α345 NC1 trimer reveals an unexpected pairing of chains at the hexamer interface. In the crystal structures of the α121 NC1, the pairing follows the rule even–even and odd–odd, that is, α2–α2 and α1–α1, which is consistent with covalent sulfilimine cross-linking of these pairs (8). Based on sulfilimine cross-linking analysis of the GBM α345 hexamer, the expected chain pairs were α3–α5 and α4–α4 (7), although in the crystal structure, we observed α3–α3 and α4–α5 pairs. Thus, the present crystal structure of the α345 hexamer demonstrates a labile nature of a trimer–trimer orientation in the hexamer before sulfilimine cross-linking. Presumably, during crystallization process, only one particular form was selected as compatible with a given crystal packing. In support of this labile nature of the hexamer are the crystal structures of α3 and α5 homohexamers, which demonstrated alternative pairs of chains in hexamers (13). How nature selects one particular orientation before covalent cross-linking by sulfilimine bonds remains to be explored. In conclusion, our single-chain NC1 trimer method allowed for determination of the α345 hexamer crystal structure, a major goal that has been pursued by scientists for decades.

### The chloride ions at the NC1 α345 trimer–trimer interface

**Figure 4:**
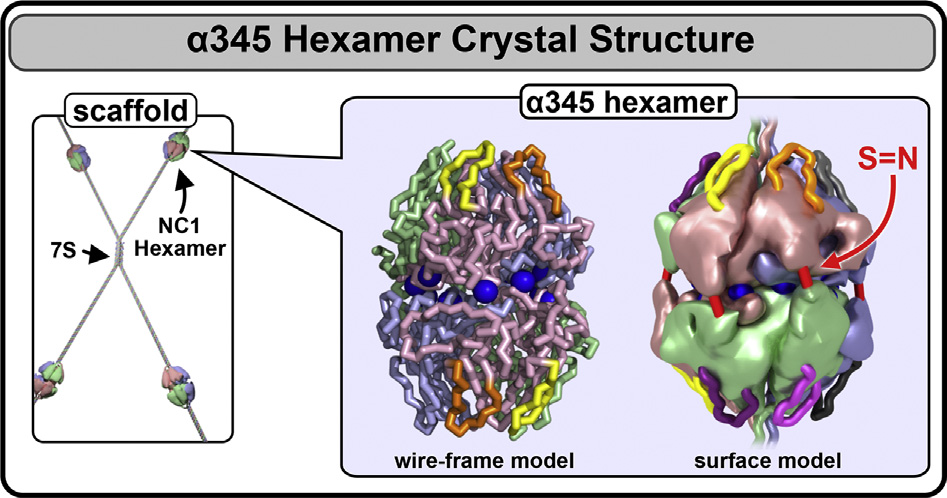
**The crystal structure of the collagen IV**^**α345**^**hexamer.** In the GBM, collagen IV^α345^ scaffold is built from α345 protomers, where the NC1 hexameric complex is a key assembly unit (*left*). The crystal structure of the α345 hexamer is shown as a backbone wire-frame (*middle*) depicting loops (GP hypoepitopes) and the chloride ring at the trimer–trimer interface. The corrected surface model (*right*) depicts the appropriate chain pairing and sulfilimine crosslinks (S = N; shown in *red*). GBM, glomerular basement membrane.

**Figure 5:**
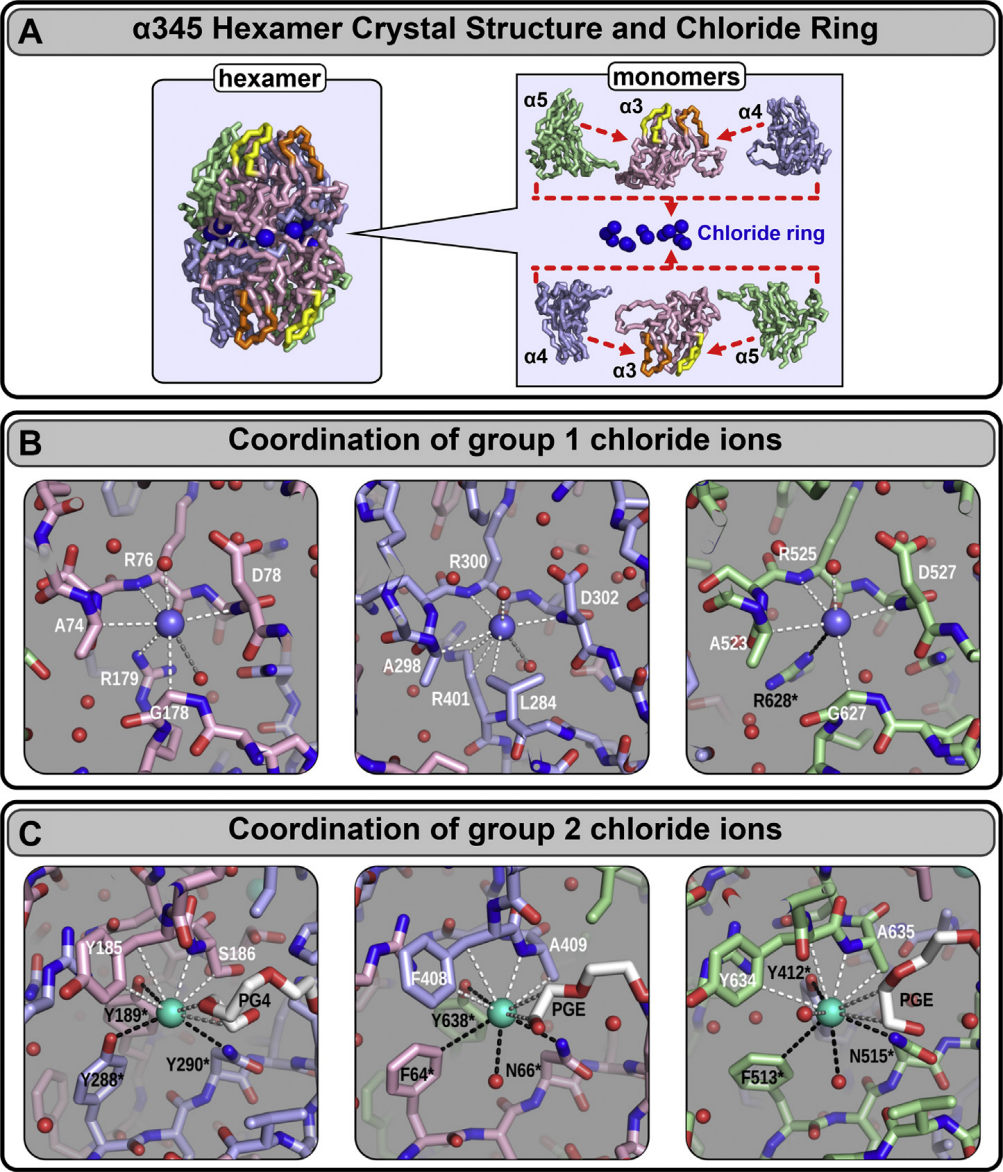
**The α345 hexamer crystal structure reveals a ring of chloride ions that coordinate the trimer–trimer interface.***A*, the crystal structure of the α345 hexamer is shown as a backbone wireframe (*left*). Individual chains are indicated by different colors as shown on the *right*. E_A_ and E_B_ loops in the α3 chain are shown in *yellow* and *orange*, respectively. 12 chloride ions (*blue spheres*) form a Cl^-^ ring at the interface between two α345 trimers (*left* and *right*). *B*, chloride ion coordination for group 1 chloride. Group 1 chloride are responsible for introducing intramolecular salt bridges utilizing chloride coordinating Arg (R76, R300, and R525 in α3, α4, and α5 respectively). Coloring for group 1 chloride is *blue* shown as *blue spheres*, while carbon atom coloring in NC1 chains is *light red* for α3, *light blue* for α4, and *light green* for α5. *C*, chloride ion coordination for group 2 chloride. Group 2 chloride are responsible for directly bridging trimeric protomers in the collagen IV hexamer, and unlike group 1 chloride, are available for interaction with PEG molecules (PG4 and PGE). Coloring for group 2 chloride is *cyan*, while carbon atom coloring in PEG molecules (PG4 and PGE) is *white*. Chloride interactions from one NC1 molecule are shown as *white dashes*, while interactions from the opposite molecule are shown as *black dashes*. Carbon atom coloring for NC1 chains is the same as above.

Despite rotational mismatch of the crystallized hexamer, we discovered a set of twelve Cl^-^ ions at the trimer–trimer interface (Fig. 4) having the same geometry as in the α121 hexamer (5). All 12 Cl^-^ at the trimer–trimer interface have comparable electron densities (Fig. S4) and atomic displacement factors in the range from 19.1 to 19.7 Å^2^, like the core residues of the polypeptide chain (Fig. 8). Together, the 12 ions form a chloride ring at the hexamer interface (Fig. 5). Analogously to the Cl^-^ ions in the α121 hexamer, these ions form two structurally different groups (Figs. 5, S4, and Table S4). Geometry and residue specificity of Cl^-^ coordination are identical between α345 and α121, with only one exception, that is, two-thirds of group 1 ions (four ions per hexamer) are not coordinated by the salt bridge to the arginine residue of the opposite trimer, rather identical arginine within the same trimer coordinates respective ions (Cl^-^ ions #1–2). Potentially, the absence of these salt bridges might impact the hexamer stability of the crystallized form. The other two Cl^-^ ions (#3) in group 1 have ‘classical’ geometry observed in the α121 hexamer with the arginine residues from the opposite trimer involved in the coordination (5).

**Figure 6:**
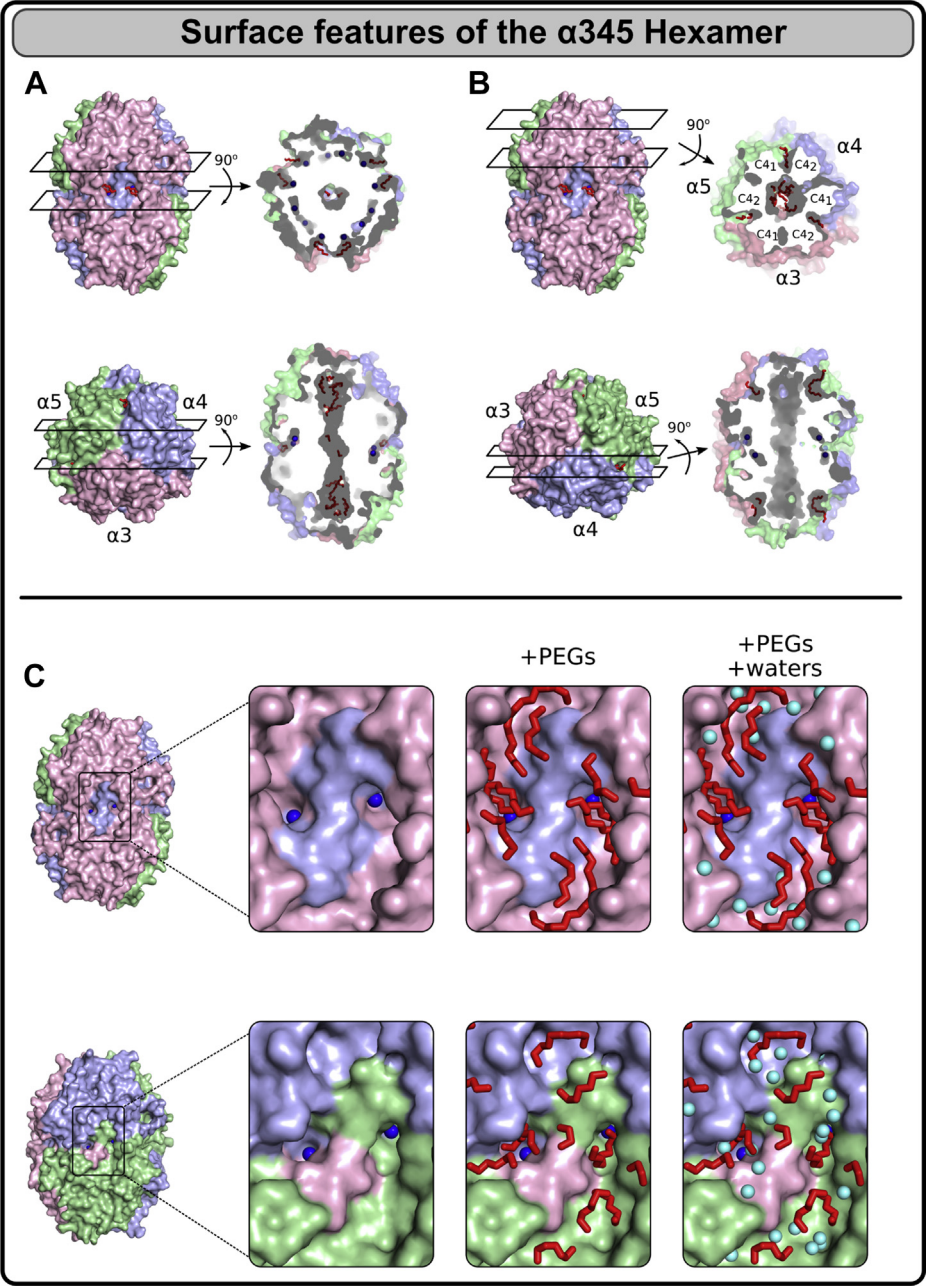
**The α345 hexamer crystal structure reveals crevices, pockets, and cavities along the surface and are solvent accessible.***A*, equatorial and meridian slices through the surface of the hexamer reveal a big central cavity going from one trimer to the other, small inner cavities encapsulating six chloride ions of group 1, and pockets accommodating six chloride ions of group 2. The central cavity contains multiple structured PEG molecules. Pockets also contain parts of PEG molecules. *B*, the same orientation of slices but shifted toward the crevices between chains and C4 subdomains. Interchain crevices are occupied by PEG molecules. Outside surface of the α345 hexamer is colored in *light red* for α3, *light blue* for α4, and *light green* for α5. The inner surface is colored in *gray*. Chloride ions of both group 1 and group 2 are shown as *blue spheres*. PEG molecules are shown as *red* wireframes. *C*, PEG molecules penetrate and surround the pockets with chloride ions. Coloring of the NC1 chains is *light red* for α3, *light blue* for α4, and *light green* for α5. Chloride ions of group 2 are shown as *blue spheres*. Structured PEG molecules are shown as *red* wireframes. Structured water molecules are shown as *cyan spheres*.

Chloride ions of group 2 are located in pockets and also interact with PEG molecules used for crystallization (Fig. 6*C* and Table S4). Although those interactions are nonspecific and weak, they point to possibility to modulate chloride binding and the hexamer assembly by specifically developed agents, which might become drugs. In summary, like the α121 hexamer, the α345 hexamer possesses two groups of chloride ions at the trimer–trimer interface forming a 12-ion ring critical for hexamer assembly and stability.

### Crevices, pockets, and inner cavities in the α345 hexamer

The crystal structure of the α345 hexamer reveals several crevices, pockets, and inner cavities, which are large enough to accommodate small molecules (Fig. 6, *A* and *B*). Under crystallization conditions used, we observe not only chloride ions, which are physiologically relevant and critical for the hexamer assembly, but also multiple PEG molecules. As discussed earlier, the chloride ions of group 2 are sitting at the bottom of pockets, which are also occupied by PEG molecules. Chloride ions of group 2 are localized in small inner cavities, which additionally contain several structured water molecules (Fig. 6*C*). The central inner cavity going from one trimer to another through the hexamer interface accommodates multiple structured PEG molecules but would accommodate much larger molecules if present during protein folding or the hexamer assembly. We also found crevices between chains and between C4 subdomains within each chain. The crevices between chains are wider and occupied by PEG molecules (Fig. 6*B*). The crevices are close enough to the inner cavity and potentially there is a communication between these structures under physiological conditions. In support of this molecular channel is the presence of PEG molecules in the inner cavity, although the protein used for crystallization has been already in the hexamer form (addressed below). Thus, even for the fully assembled hexamer there is a mechanism of penetration of ligands into the inner cavity.

The outer surface of the α345 hexamer represents a complex landscape with multiple hills and valleys. We found multiple PEG molecules interacting with the surface and some of them having contacts with two adjacent chains (Fig. S5).

### GP hypoepitope loops on the surface of the α345 hexamer

**Figure 7:**
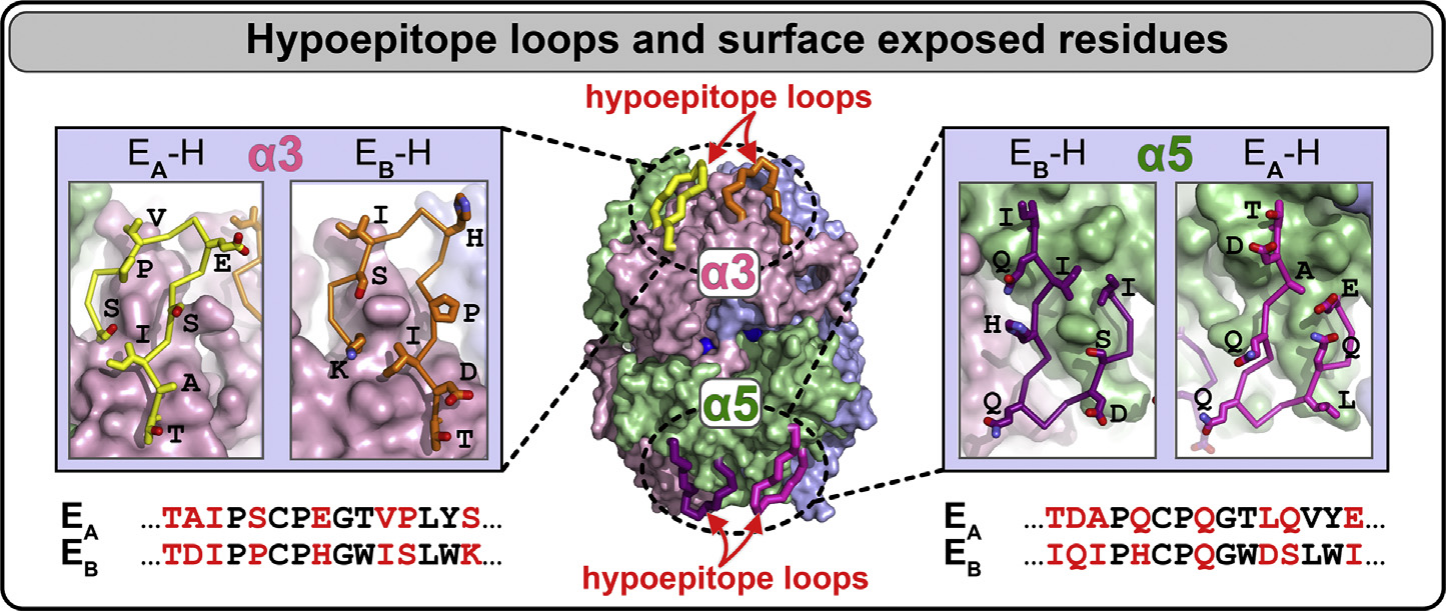
**The α345 hexamer crystal structure reveals the E**_**A**_**and E**_**B**_**loops on the surface of the α3 and α5 NC1 domains within the native α345NC1 hexamer.** The loops are presented in different colors as indicated. Insets show the side-chain geometry for surface residues available for signaling and binding with other partners. The corresponding loop sequences are shown at the bottom with surface residues highlighted in *red font.*

**Figure 8:**
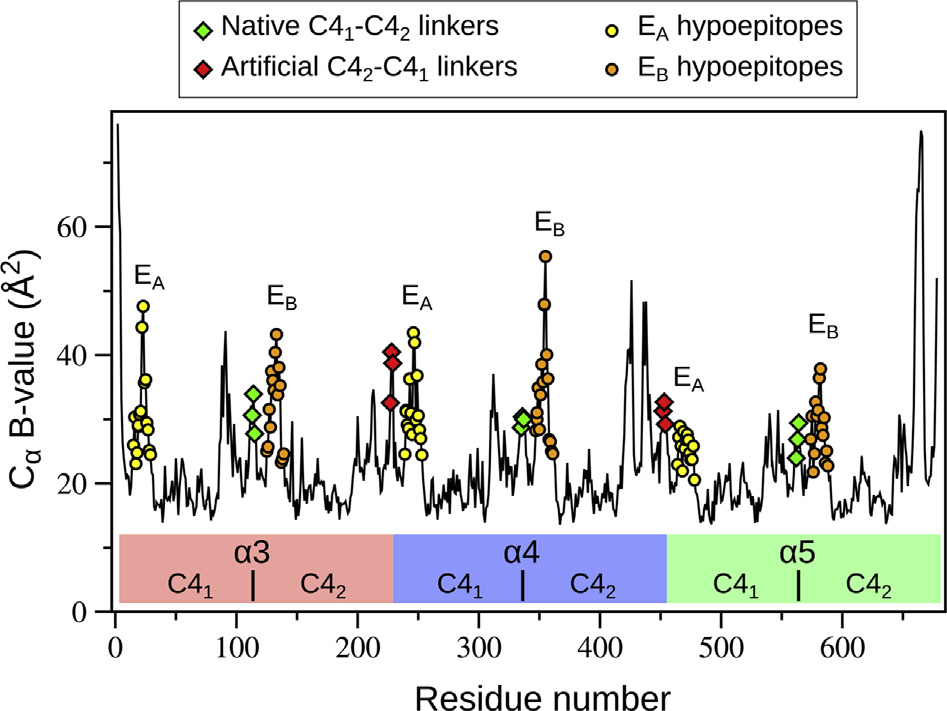
**Mean square displacement (B values) of Cα atoms.** B values for native and artificial linkers are comparable as depicted by *green* and *red diamonds*, respectively. B values for E_A_ and E_B_ hypoepitopes are shown as *yellow* and *orange circles*. Residue positions are marked with colored bars: *light red* for α3, *light blue* for α4, and *light green* for α5 chains. Borders between C4_1_ and C4_2_ subdomains are depicted as *vertical lines*.

There are surface-exposed loops on the α345 hexamer (Fig. 7) that encompass the E_A_ and E_B_ regions of GP immunoreactivity (14, 15). The loops are designated herein as hypoepitopes as they are not recognized by GP autoantibodies but can undergo a conformational transition into neoepitopes that bind the antibodies (16, 17). The E_A_ and E_B_ loops (Fig. 7) demonstrate elevated mean square displacement values (Fig. 8), which reflect increased dynamic mobility of the loops. The E_A_ loop of α5 chain is involved in crystal packing, thus having relatively lower B values (Fig. 8). Mutation analysis showed evolutionary pressure on the loop sequences, particularly on the E_A_ loop in α2-α5 chains (Fig. S6), supporting the functional importance of these loops. One of the crevices is located between E_A_ and E_B_ hypoepitope loops forming loop-crevice-loop (LCL) regions at the apexes of the α345 hexamer and is juxtaposed with the T-cell receptor epitopes (18, 19, 20).

### Z-appendage location within the α345 hexamer

**Figure 9:**
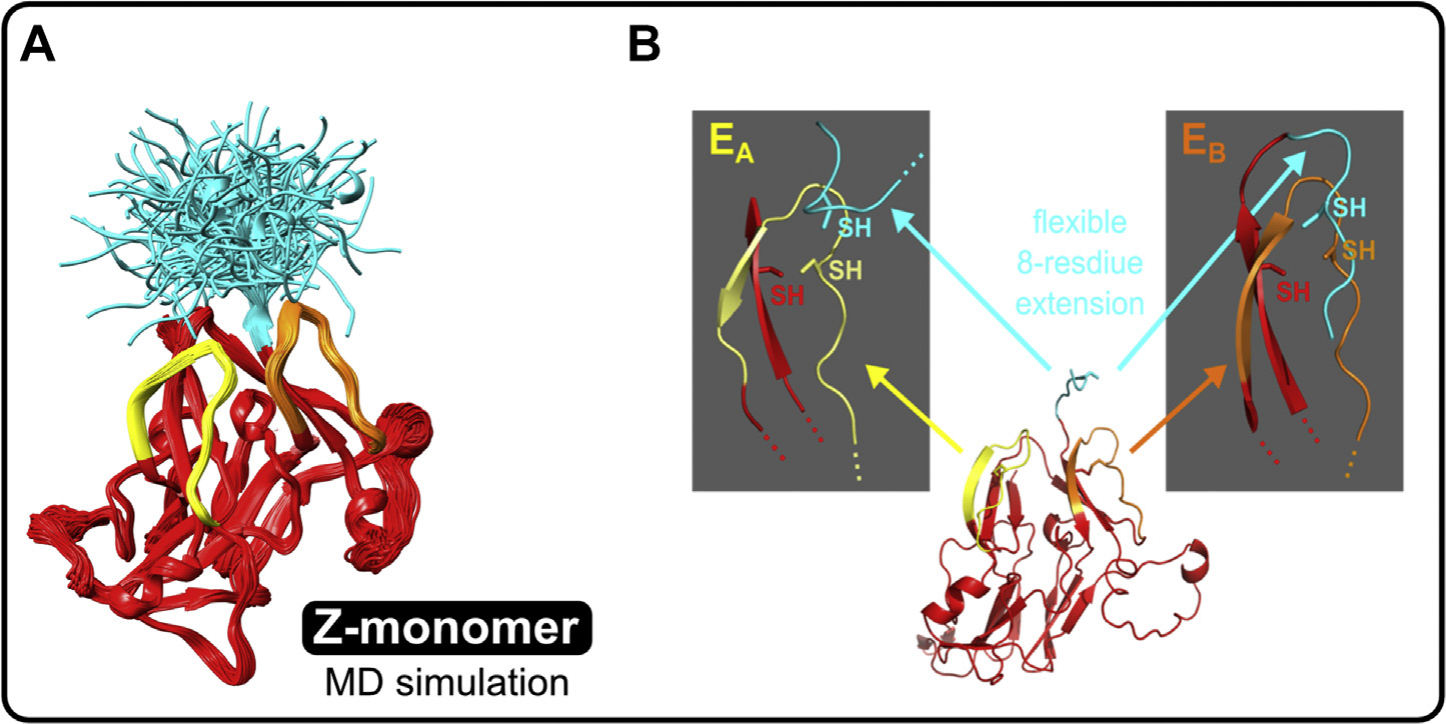
**Molecular dynamic (MD) simulations predict the Z-appendage can assume multiple conformations.***A*, a molecular dynamics simulation (1000 independent runs) (*cyan*) was performed to sample conformations of the Zurich variant. *B*, MD simulation analysis revealed two clusters of conformants of the 8-residue extension particularly close to the E_A_ or E_B_ regions. This confirms that free cysteine residue within the extension can form alternative disulfide bridges and disturb other interactions within the monomer. Interference with disulfide formation can lead to conformational changes of the E_A_ and E_B_ regions leading to appearance of immunogenic neoepitopes.

The Z-appendage is an 8-residue C-terminal extension of the native primary structure of the α3 chain of collagen IV. It is located at the apex of the α3NC1 monomer, in juxtaposition with E_A_ and E_B_ hypoepitope loops (Fig. 9). To assess Z-appendage flexibility, a molecular dynamics (MD) simulation was performed on the appendage in the context of a model of the α3 chain NC1 monomer. To sample all possible orientations of the appendage, 1000 initial conformations were originally generated by high-temperature MD. Each of those was extended for an additional 1 ns of simulation time at the physiological temperature, resulting in 1 μs of total MD sampling. Cysteines were reduced. Clustering analysis of the Z-appendage residues from all 1000 trajectories (using a 7 Å r.m.s.d. cutoff) revealed 134 conformational families. Within two of those 134 clusters, we found multiple conformations of the Z-appendage that had the cysteine residue positioned adjacent to the E_A_ or E_B_ loop disulfides. Two such conformations are shown in Figure 9, *B* and *C*. In these two conformations, the mutant cysteine is near the WT E_A_/E_B_ cysteines that form an intraloop disulfide. These conformations suggest that the appendage cysteine residue may form alternative disulfides. Interference with disulfide formation and disturbing other interactions within the monomer can lead to a conformational change of the monomer and influence assembly of the hexamer. In conclusion, the Z-appendage can assume multiple conformations and its free thiol group can participate in a number of reactions including those with E_A_ and E_B_ epitope loops.

### Analysis of known Alport variants in COL4A3, COL4A4, and COL4A5

**Figure 10:**
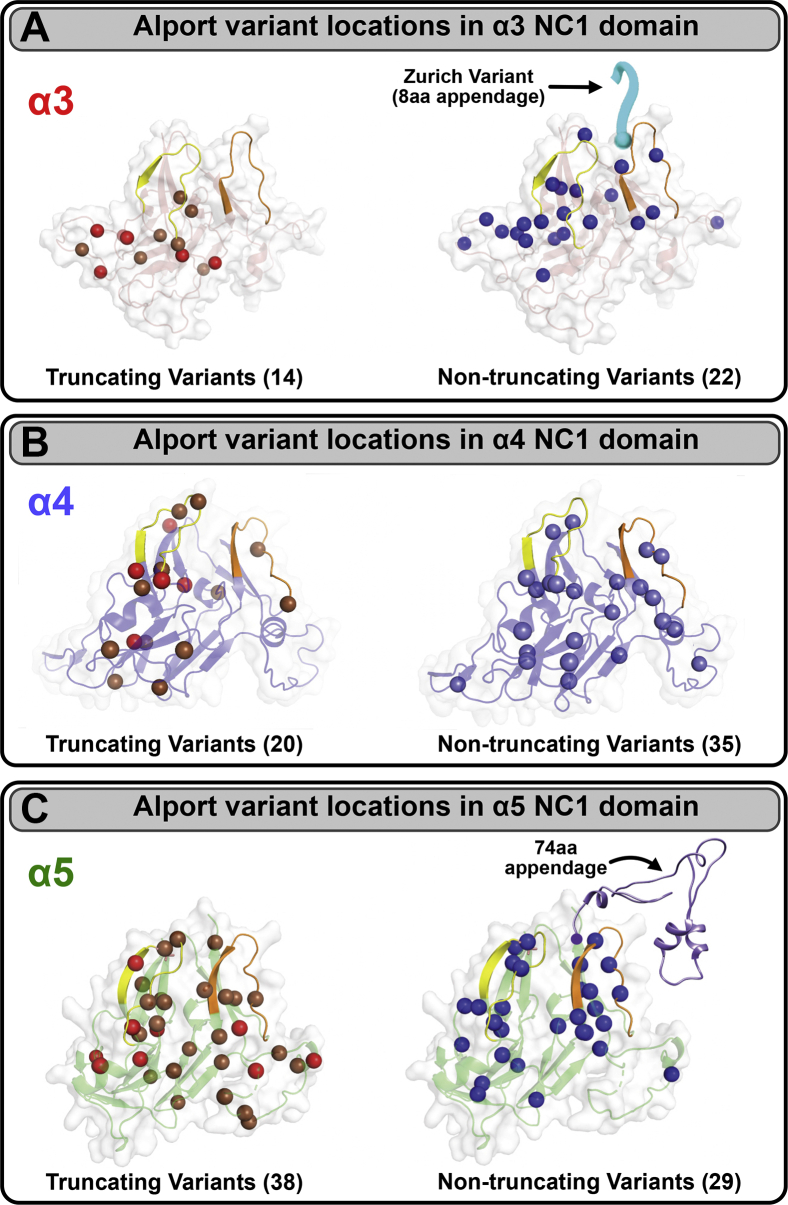
**Crystal structure of the α345 hexamer reveals the location of known Alport variants in the α3, α4, and α5 NC1 domains.***A*, distinct pathogenic Alport variants were mapped within the 3D structure of the α3 NC1 domain (source: HGMD 2020.1). The total number of truncating (small deletion, *brown*; nonsense, *red*) and nontruncating (missense variants, *blue*) variants are shown in parentheses. Truncating variants result in the premature stop codon and expression of the truncated form of COL4A3, which does not incorporate into the GBM (*left*). Although nontruncating missense variants (*blue*) do not affect the overall length of the NC1 domain (*right*), they may result in conformational changes of crucial regions within NC1. These nontruncating variants can incorporate into the GBM but are functionally defective. The Zurich variant (*cyan*) is a combination of a small deletion and insertion, resulting in an 8-aa appendage to the NC1 domain. Therefore, it belongs to “nontruncating” variant subgroup (*right*). *B*, distinct pathogenic Alport variants were mapped within the 3D structure of the α4 NC1 domain (source: HGMD 2020.1). Color codes are as in panel A. *C*, distinct pathogenic Alport variants were mapped within the 3D structure of the α5 NC1 domain (source: HGMD 2020.1). A distinct Alport variant, 74-amino acid appendage at the C-terminus of α5 chain, is depicted on the right. Color codes are as in *panel A*. For an expanded description of the genetic identity of the α3, α4, and α5 variants, see Figs. S12–S14. GBM, glomerular basement membrane.

Solving the crystal structure of the α345 hexamer allowed for 3D mapping of known Alport variants. The maps of Alport variants in the α3, α4, and α5 chains of collagen IV and their localizations within 3D structures of the α3, α4, and α5NC1 domains are shown in Figure 10 (for additional details, see Figs. S7–S9). The analysis reveals two classes of NC1 variants, that is, truncating and nontruncating. Potentially, both classes are amenable to protein replacement therapy and the nontruncating class also presents a possibility for development of small-molecule therapies. The descriptions for each variant are provided in the top part of Figs. S7–S9, according to the human genome variation society nomenclature (21). The Zurich variant of the α3NC1 domain stands out among other Alport variants as it results in a C-terminal extension of protein polypeptide chain producing an 8-amino acid Z-appendage as shown in Fig. S7; a variant analogous to the Zurich variant producing a 74-amino acid appendage has been identified in α5NC1 (Fig. S9).

### Analysis of potential glycoxidation sites on the surface of the α345 hexamer relevant to DN

**Figure 11:**
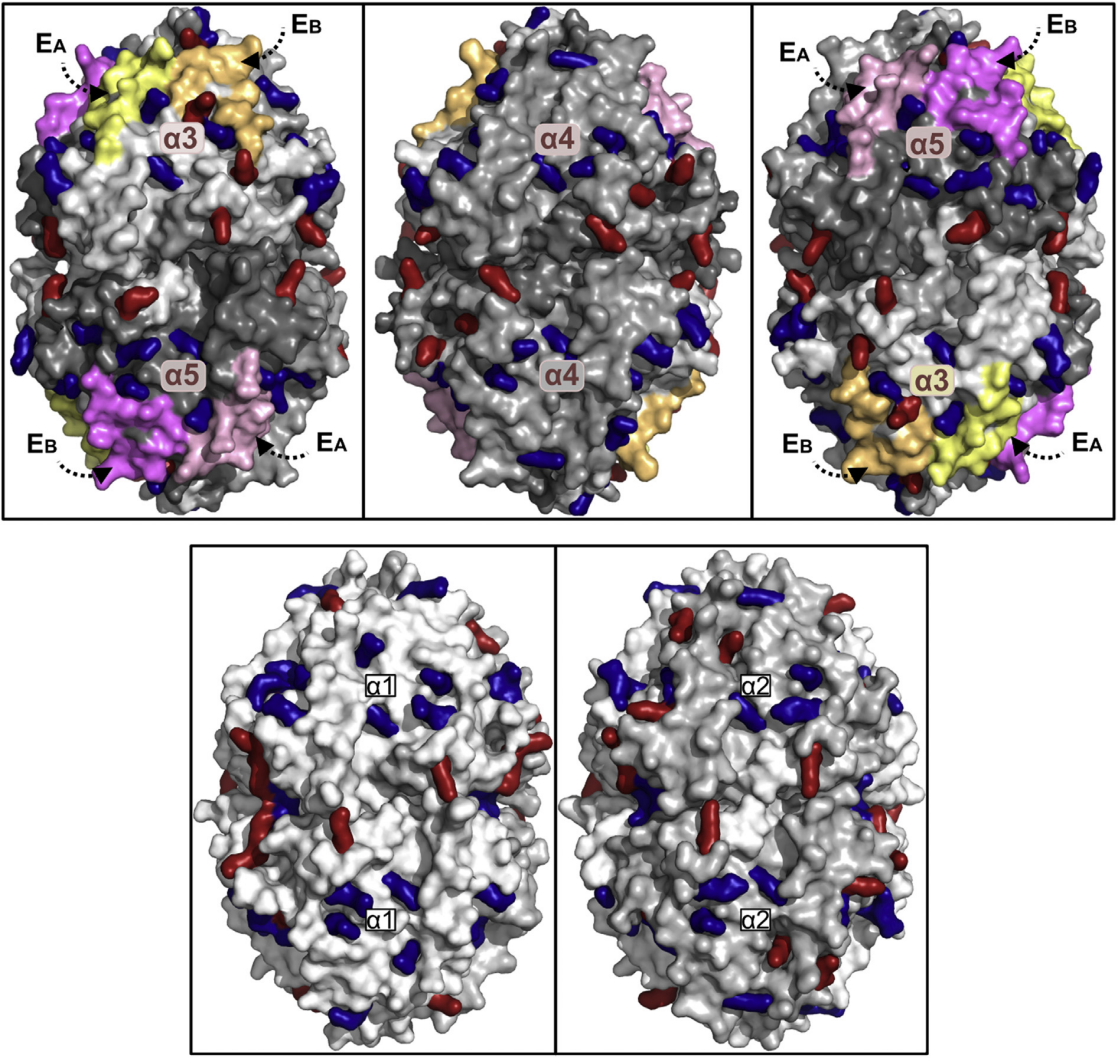
**The surface-exposed lysine (Lys) and arginine (Arg) residues of collagen IV hexamers of α345 (*top*) and α121 (*bottom*) GBM scaffolds that can be adducted by glucose and glucose-derived reactive carbonyl products in diabetes.***Top panel*: The 3D structure of α345 hexamer is shown in three different projections rotated by 120° about the vertical axis. The labels indicate individual α3, α4, and α5 NC1 domains within the hexamer structure and locations of E_A_ and E_B_ hypoepitopes. The surface-exposed Lys and Arg side chains are shown in *red* and *blue colors*, respectively. *Bottom panel*: the 3D structure of α121 hexamer in two projections (120° rotation about the vertical axis) showing the location of the surface-exposed Lys and Arg side chains using the same color coding as in the *top* panel. GBM, glomerular basement membrane.

The α345 hexamer possesses multiple surface-exposed lysine (Lys) and arginine (Arg) residues (Fig. 11) that can be targeted by hyperglycemia-derived reactive carbonyl products to form stable adducts that underlie DN pathogenesis, including Lys–Lys and Lys–Arg crosslinks (22). There are 78 surface-exposed Lys and Arg side chains in the hexamer. Importantly, six of these residues in the α3NC1 domain and four in the α5NC1 domain are adjacent to or located on the respective E_A_ and E_B_ hypoepitopes (Fig. 11).

## Discussion

**Figure 12:**
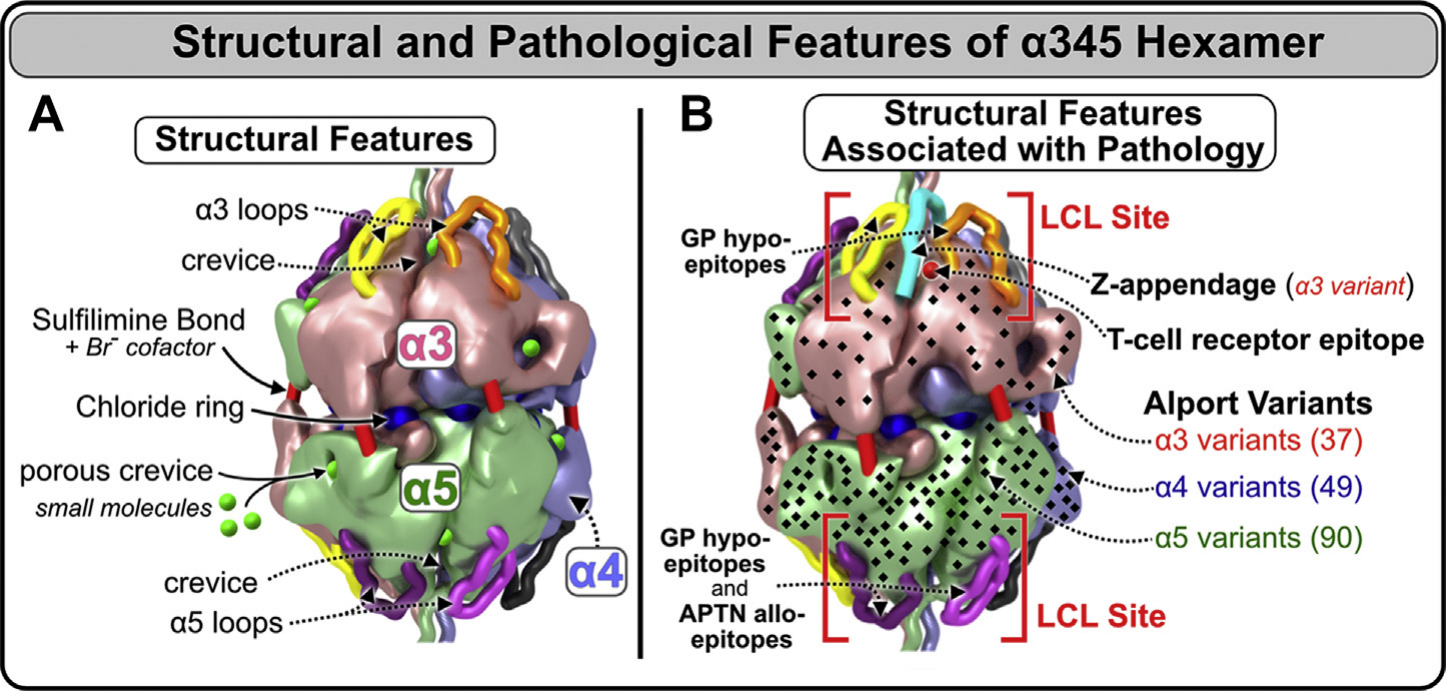
**Structural and pathological features of the α345 hexamer.***A*, the α345 hexamer is a key connection module within the collagen IV^α345^ scaffold. The hexamer structure features a ring of 12 chloride ions, required for hexamer assembly, at the interface of the two trimers. The assembled hexamer also features up to six sulfilimine bonds between the protomers that reinforce the stability of the scaffold. The surface of α345 hexamer is marked by multiple pores and crevices that are accessible to small molecules. *B*, the α345 hexamer harbors a number of features involved in pathogenesis of Alport syndrome and Goodpasture's diseases. Multiple Alport-associated variants occur within α3, α4, and α5 NC1 domains (*black dots*) including the Zurich variant of α3 NC1, which produces an 8-amino acid Z-appendage shown in *cyan*. In juxtaposition to the Z-appendage, there are Goodpasture's disease (GP) hypoepitope loops and a T-cell receptor epitope located at the bottom of the crevice between the loops. Together, these features constitute a loop-crevice-loop (LCL) site where pathogenic mechanism of Alport syndrome and Goodpasture diseases converge (*top red square brackets*). The analogous LCL site is located within the α5 NC1 domain (*bottom red square brackets*) and within the α4 NC1 domain (*not shown*).

The crystal structure of the α345 hexamer provided a framework to interpret a role that the Z-appendage, a representative AS variant, played in AS and as a possible structural risk factor for GP, as described in Pokidysheva *et al*. (4). The crystal structure of the hexamer revealed a ring of 12 chloride ions that, together with up to six sulfilimine bonds, stabilizes the hexamer structure (Fig. 12*A*). The α345 hexamer harbors a number of structural features associated with pathology, which are located within the LCL sites where pathogenic mechanisms of AS and GP converge, and potentially DN. Within the LCL sites, there are multiple Alport-associated variants in the α3, α4, and α5 NC1 domains including the Z-appendage, which is juxtaposed with the GP hypoepitopes (Fig. 12*B*). In addition, GP hypoepitope loops and a T-cell receptor epitope (18, 19, 20) are located within the LCL sites (Fig. 12*B*).

**Figure 13:**
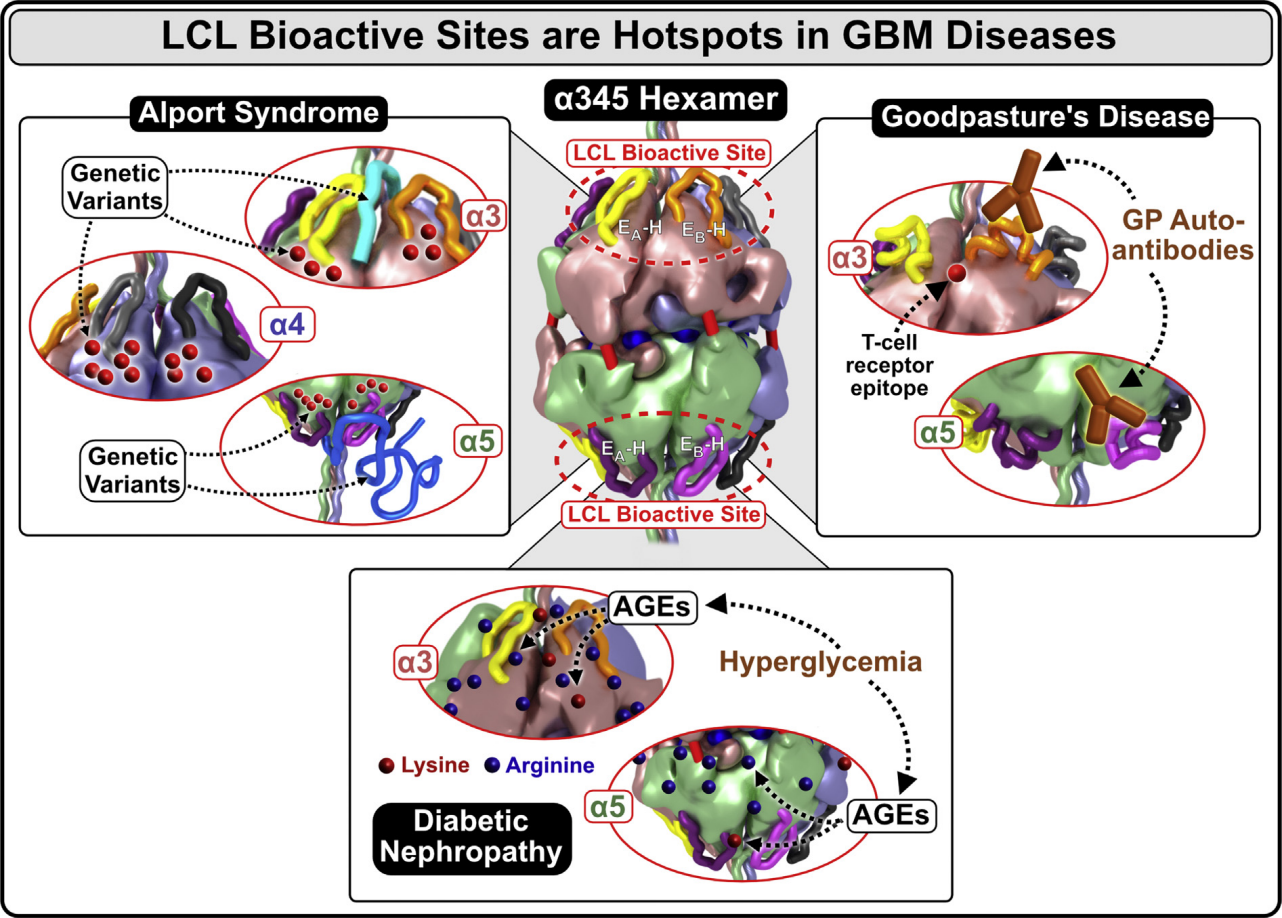
**The crystal structure of the α345 hexamer reveals common “hotspots” of bioactivity, where pathogenic mechanisms converge.** The Z-appendage and the GP hypoepitopes are located at the same sites of α3 and α5 subunits near the apices of the α345 hexamer which are called loop-crevice-loop (LCL) bioactive sites (α4 LCL site located on *back side* of the hexamer). This indicates that the pathogenic mechanisms of GP and AS converge at these sites, thus revealing “hotspots” of bioactivity. In Alport syndrome, a number of pathogenic variants including the Zurich variant are located within the LCL sites (*top left*). In addition, there are numerous other hypomorph variants on the surface that can affect LCL function (see Figs. S12–S14). In familial GP disease, the Zurich variant within the LCL sites could predispose the site for a trigger of autoantibody production (*top right*). In sporadic GP, the same LCL site is vulnerable to endogenous and exogenous triggers that elicit the immune response. Similarly, in diabetic nephropathy, this site is vulnerable to hyperglycemia-derived modifications of Lys and Arg residues at the hexamer surface. These modifications are in fact equivalent to genetic variants that cause structural perturbation and dysfunction. They can cause GBM thickening, a hallmark feature of diabetic nephropathy (35). AS, Alport syndrome; GBM, glomerular basement membrane; GP, Goodpasture’s disease.

Furthermore, the 3D structure provided the framework for designing hexamer assembly studies in Pedchenko *et al*. (23), which demonstrated that the LCL sites have conformational plasticity. This plasticity along with the structural features of the LCL sites indicate bioactive functions that may include signaling and organizing macromolecular complexes. These functions can be perturbed by the Z-appendage and other genetic variants that occur in the hexamer in AS, endogenous and exogenous triggers in GP, and hyperglycemia in DN (Fig. 13). Moreover, because a significant number of Alport variants occurs throughout the hexamer structure (Fig. 12), the multiple pores, crevices, and cavities on the surface of the hexamer can be potential targets for therapeutic interventions such as small-molecule drugs and protein replacement.

## Experimental procedures

### Design, expression, and purification of single-chain NC1 trimers

Four combinations, α345, α543, α343, and α545, of human DNA sequences encoding residues of α3, α4, and α5 of collagen IV NC1 domain were cloned in-frame with the BM-40 signal peptide and the FLAG tag of the pRc-X vector (14) between restriction sites NheI and BspDI using the previously developed strategy (5). Constructs were transiently expressed (and, in case of α343 and α545, coexpressed) in ExpiCHO cells. For the single-chain α345 NC1 trimer construct, a stable clone of HEK293 cells was developed as described (5) and used for bulk production of the α345 NC1 trimer for assembly studies and crystallization. The recombinant protein fused with an N-terminal FLAG tag was purified using FLAG-affinity resin as described (6). Size-exclusion chromatography using Superdex 200 Increase 10/300GL column (GE Healthcare) was used for the final purification step.

### Single-chain α345 NC1 trimer crystallization and structure determination

The single-chain α345 NC1 trimer was crystallized in the tetragonal form (space group, P4_1_2_1_2) using the hanging-drop vapor diffusion method. The protein solution (∼10 mg/ml) in 5-mM Tris HCl, pH 7.5, and 150-mM NaCl was mixed for the drop solution in a 1:1 proportion with a reservoir solution of 100-mM Tris HCl, pH 8.5, and 56% PEG 200. The crystals grew to a final size of ∼0.2 × 0.15 × 0.15 mm after 45 days at 22 °C. The crystals were flash-frozen in liquid nitrogen. Data collection was performed remotely on crystals cryocooled to 100 K at the Life Sciences Collaborative Access Team beamline 21-ID-G at the Advanced Photon Source, Argonne National Laboratory. Data extending to 1.85 Å resolution were indexed using iMOSFLM (24) and then scaled and merged using Scala (25). Amplitudes were converted to structure factors using CTRUNCATE (26). Five percent of the data were set aside to monitor *R*_free_. Initial phases were obtained by molecular replacement using Phaser-MR (27) and the previously solved single-chain α112 NC1 trimer (PDB code: 6MPX) (5) as the search model. One single-chain NC1 polypeptide was found per asymmetric unit (*V*_*M*_ = 2.82 Å^3^/Da; solvent content = 56.4% (28)). Refinement was carried out using Phenix (29) with translation–libration–screw-rotation model restraints. The models were manually adjusted between each refinement cycle using Coot (30). Model geometry assessed using MolProbity (31) showed 97.5% of the residues in the favored region and 2.5% in the additionally allowed region, with none in the outlier regions. The final data collection and refinement statistics are shown in Table S6. Model superimpositions were performed using LSQ Superpose function in Coot (30).

### Structural modeling of the NC1 domain: MD simulations

To examine the conformational space accessible by the mutant residues, we performed 1000 1-ns MD simulations of the mutant α3 monomer using the graphics processing unit-enabled codes in the AMBER18 suite of molecular mechanics programs (32). We began by extracting the monomer structure from a previously published α345 model (33) and from the structure solved in the present study. The mutant residues, in an extended strand-like conformation, were appended to the WT residues. We then heated this extended mutant to 1000K while holding the WT residues close to their starting position using restraints and modeling the cysteine residues in a reducing environment. We used the generalized born (GB) implicit solvent model to maximize conformational kinetics by removing friction with solvent molecules while still providing a good approximation of solvent-shielding effects (34). We then performed a 10-ns restrained MD simulation at 1000K from which we captured a snapshot every 10 ps. The resulting 1000 snapshots were then cooled to 310 K over 50 ps and used as the starting structures for 1000 x 1-ns unrestrained MD simulations at 310 K, also using the GB approximation. The resulting 1 μs worth of MD conformational samples were then clustered and analyzed with the CPPTRAJ program, to visualize representative conformational accessibility and to analyze distances between the mutant cysteine and the WT cysteine CG atoms.

## Data availability

All data described in this article are available in the main text or supporting information. The atomic coordinates and structure factors (code 6WKU) have been deposited in the Protein Data Bank (http://wwpdb.org/).

## Supporting information

This article contains supporting information (13, 36)

## Conflict of interest

The authors declare that they have no conflicts of interest with the contents of this article.
