# Supplementary material for: Collagen IVα345 dysfunction in glomerular basement membrane diseases. II. Crystal structure of the α345 hexamer
Source: J Biol Chem. 2021 Mar 26;296:100591. doi: 10.1016/j.jbc.2021.100591 (PMC8093946; doi:10.1016/j.jbc.2021.100591)
Supplement: Supplementary Material [file mmc1.pdf]

SUPPLEMENTARY APPENDIX

This appendix has been provided by the authors to give readers additional information about their work.

Supplement to: Mechanisms of Collagen IV345 assembly and dysfunction in Goodpasture's and Alport diseases: II. Crystal structure of a345 hexamer reveals bioactive sites.

Table of Contents

	Page
**Supplementary Section 1:** ***Composition and arrangement of chains within the α345 hexamer*** (*Supplementary Figs. 1 and 2*)	2
**Supplementary Section 2:** ***Crystal structure of the NC1 α345 hexamer*** (*Supplementary Fig. 3, Supplementary Tables 1-3*)	4
**Supplementary Section 3:** ***The chloride ions at the NC1 α345 trimer-trimer interface*** (*Supplementary Fig. 4, Supplementary Table 4*)	7
**Supplementary Section 4:** ***Crevices, pockets, inner cavities, loops and surface-exposed reactive residues in the α345 hexamer*** (*Supplementary Figs. 5-6*)	9
**Supplementary Section 5:** ***Analysis of known Alport variants in COL4A3, COL4A4, and COL4A5*** (*Supplementary Figs. 7-9, Supplementary Tables 5 and 6*)	11

Supplementary Section 1: Composition and arrangement of chains within the α345 hexamer

>human_a345NC1

MRAWIFFLLCLAGRALAAPLADYKDDDDKLAGATWTTRGFVFTRHSQTTAIPSCPEGTVPLYSGFSFLFVQGNQRAH
GQDLGTLGSCLQRFTTMPFLFCNVNDVCNFASRNDYSYWLSTPALMPMNMAPITGRALEPYISRCTVCEGPAIAIAV
HSQTTDIPPCPHGWISLWKGFSFIMFTSAGSEGTGQALASPGSCLEEFRASPFLECHGRGTCNYYSNSYSFWLASLN
PERMFRKPIPSTVKAGELEKIISRCQVCMGTGFLLVLHSQTDQEPTCPLGMPRLWTGYSLLYLEGQEKAHNQDLGLA
GSCLPVFSTLPFAYCNIHQVCHYAQRNDRSYWLASAAPLPMMPLSEEAIRPYVSRCAVCEAPAQAVAVHSQDQSIPP
CPQTWRSLWIGYSFLMHTGAGDQGGGQALMSPGSCLEDFRAAPFLECQGRQGTCHFFANKYSFWLTTVKADLQFSSA
PAPDTLKESQAQRQKISRCQVCVAPGFLITRHSQTTDAPQCPQGTLQVYEGFSLLYVQGNKRAHGQDLGTAGSCLRR
FSTMPFMFCNINNVCNFASRNDYSYWLSTPEPMPMSMQPLKGQSIQPFISRCAVCEAPAVVIAVHSQTIQIPHCPQG
WDSLWIGYSFMMHTSAGAEGSGQALASPGSCLEEFRSAPFIECHGRGTCNYYANSYSFWLATVDVSDMFSKPQSETL
KAGDLRTRISRCQVCMKRT*

>human_a543NC1

MRAWIFFLLCLAGRALAAPLADYKDDDDKLAGTSSVAHGFLITRHSQTTDAPQCPQGTLQVYEGFSLLYVQGNKRAH
GQDLGTAGSCLRRFSTMPFMFCNINNVCNFASRNDYSYWLSTPEPMPMSMQPLKGQSIQPFISRCAVCEAPAVVIAV
HSQTIQIPHCPQGWDSLWIGYSFMMHTSAGAEGSGQALASPGSCLEEFRSAPFIECHGRGTCNYYANSYSFWLATVD
VSDMFSKPQSETLKAGDLRTRISRCQVCMGTGFLLVLHSQTDQEPTCPLGMPRLWTGYSLLYLEGQEKAHNQDLGLA
GSCLPVFSTLPFAYCNIHQVCHYAQRNDRSYWLASAAPLPMMPLSEEAIRPYVSRCAVCEAPAQAVAVHSQDQSIPP
CPQTWRSLWIGYSFLMHTGAGDQGGGQALMSPGSCLEDFRAAPFLECQGRQGTCHFFANKYSFWLTTVKADLQFSSA
PAPDTLKESQAQRQKISRCQVCVAPGFVFTRHSQTTAIPSCPEGTVPLYSGFSFLFVQGNQRAHGQDLGTLGSCLQR
FTTMPFLFCNVNDVCNFASRNDYSYWLSTPALMPMNMAPITGRALEPYISRCTVCEGPAIAIAVHSQTTDIPPCPHG
WISLWKGFSFIMFTSAGSEGTGQALASPGSCLEEFRASPFLECHGRGTCNYYSNSYSFWLASLNPERMFRKPIPSTV
KAGELEKIISRCQVCMKKRH*

>human_a343NC1

MRAWIFFLLCLAGRALAAPLADYKDDDDKLAGATWTTRGFVFTRHSQTTAIPSCPEGTVPLYSGFSFLFVQGNQRAH
GQDLGTLGSCLQRFTTMPFLFCNVNDVCNFASRNDYSYWLSTPALMPMNMAPITGRALEPYISRCTVCEGPAIAIAV
HSQTTDIPPCPHGWISLWKGFSFIMFTSAGSEGTGQALASPGSCLEEFRASPFLECHGRGTCNYYSNSYSFWLASLN
PERMFRKPIPSTVKAGELEKIISRCQVCMGTGFLLVLHSQTDQEPTCPLGMPRLWTGYSLLYLEGQEKAHNQDLGLA
GSCLPVFSTLPFAYCNIHQVCHYAQRNDRSYWLASAAPLPMMPLSEEAIRPYVSRCAVCEAPAQAVAVHSQDQSIPP
CPQTWRSLWIGYSFLMHTGAGDQGGGQALMSPGSCLEDFRAAPFLECQGRQGTCHFFANKYSFWLTTVKADLQFSSA
PAPDTLKESQAQRQKISRCQVCVAPGFVFTRHSQTTAIPSCPEGTVPLYSGFSFLFVQGNQRAHGQDLGTLGSCLQR
FTTMPFLFCNVNDVCNFASRNDYSYWLSTPALMPMNMAPITGRALEPYISRCTVCEGPAIAIAVHSQTTDIPPCPHG
WISLWKGFSFIMFTSAGSEGTGQALASPGSCLEEFRASPFLECHGRGTCNYYSNSYSFWLASLNPERMFRKPIPSTV
KAGELEKIISRCQVCMKKRH*

>human_a545NC1

MRAWIFFLLCLAGRALAAPLADYKDDDDKLAGTSSVAHGFLITRHSQTTDAPQCPQGTLQVYEGFSLLYVQGNKRAH
GQDLGTAGSCLRRFSTMPFMFCNINNVCNFASRNDYSYWLSTPEPMPMSMQPLKGQSIQPFISRCAVCEAPAVVIAV
HSQTIQIPHCPQGWDSLWIGYSFMMHTSAGAEGSGQALASPGSCLEEFRSAPFIECHGRGTCNYYANSYSFWLATVD
VSDMFSKPQSETLKAGDLRTRISRCQVCMGTGFLLVLHSQTDQEPTCPLGMPRLWTGYSLLYLEGQEKAHNQDLGLA
GSCLPVFSTLPFAYCNIHQVCHYAQRNDRSYWLASAAPLPMMPLSEEAIRPYVSRCAVCEAPAQAVAVHSQDQSIPP
CPQTWRSLWIGYSFLMHTGAGDQGGGQALMSPGSCLEDFRAAPFLECQGRQGTCHFFANKYSFWLTTVKADLQFSSA
PAPDTLKESQAQRQKISRCQVCVAPGFLITRHSQTTDAPQCPQGTLQVYEGFSLLYVQGNKRAHGQDLGTAGSCLRR
FSTMPFMFCNINNVCNFASRNDYSYWLSTPEPMPMSMQPLKGQSIQPFISRCAVCEAPAVVIAVHSQTIQIPHCPQG
WDSLWIGYSFMMHTSAGAEGSGQALASPGSCLEEFRSAPFIECHGRGTCNYYANSYSFWLATVDVSDMFSKPQSETL
KAGDLRTRISRCQVCMKRT*

Supplementary Fig. 1. *Amino acid sequences of single-chain NC1 trimers of human collagen IV composed from α3, α4, and α5 NC1 domains that were used in this study.* BM40 signal peptide sequences are underlined and FLAG-tag sequences are highlighted. Artificially introduced linkers GTG and APG between chains are shown in bold. NC1 domain coloring is **red for α3**, **blue for α4**, and **green for α5**.

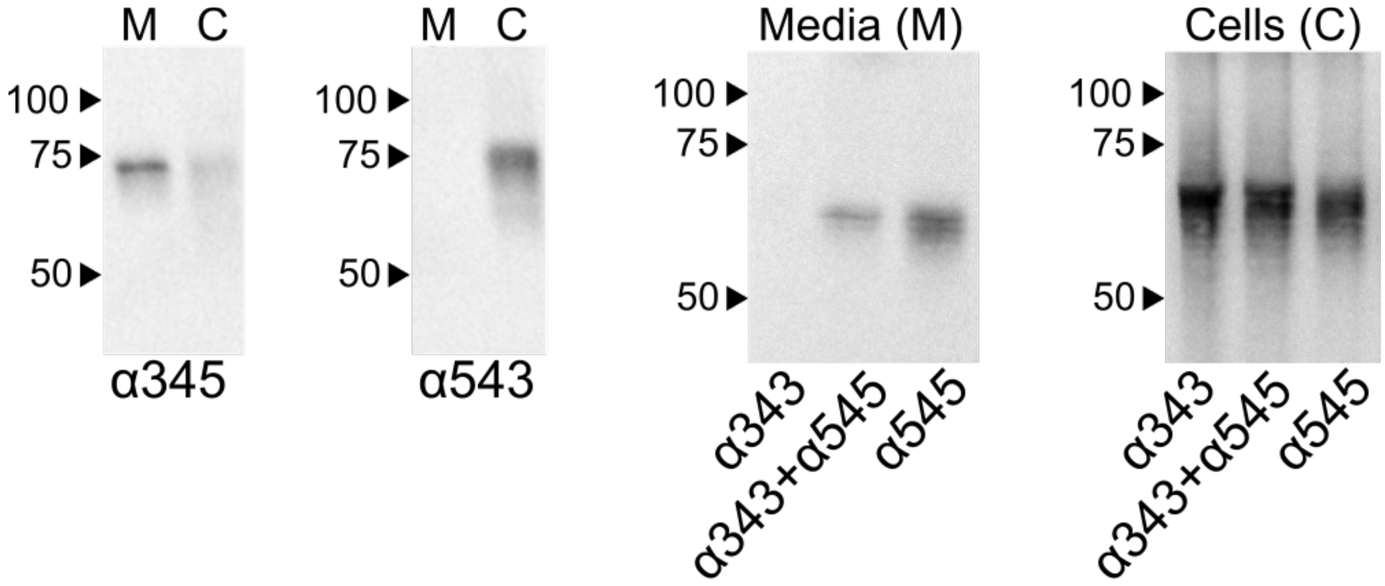

Supplementary Fig. 2. *Protein secretion analysis of different single-chain NC1 trimers using transient expression in ExpiCHO cells*. Media (M) and cell lysate (C) were analyzed for protein presence using Western blotting with JK2 antibody. Only the single-chain α345 NC1 trimer was efficiently secreted into the medium. The α543 and α345 were not detected in the medium indicating potential folding problem. The α545 showed only limited secretion to the medium, also indicating potential folding problem. Co-expression of α343 and α545 did not resolve the folding problem, ultimately excluding these combinations as native forms. Finally, only the α345 trimer present a native form.

Supplementary Section 2: Crystal structure of the α345 hexamer

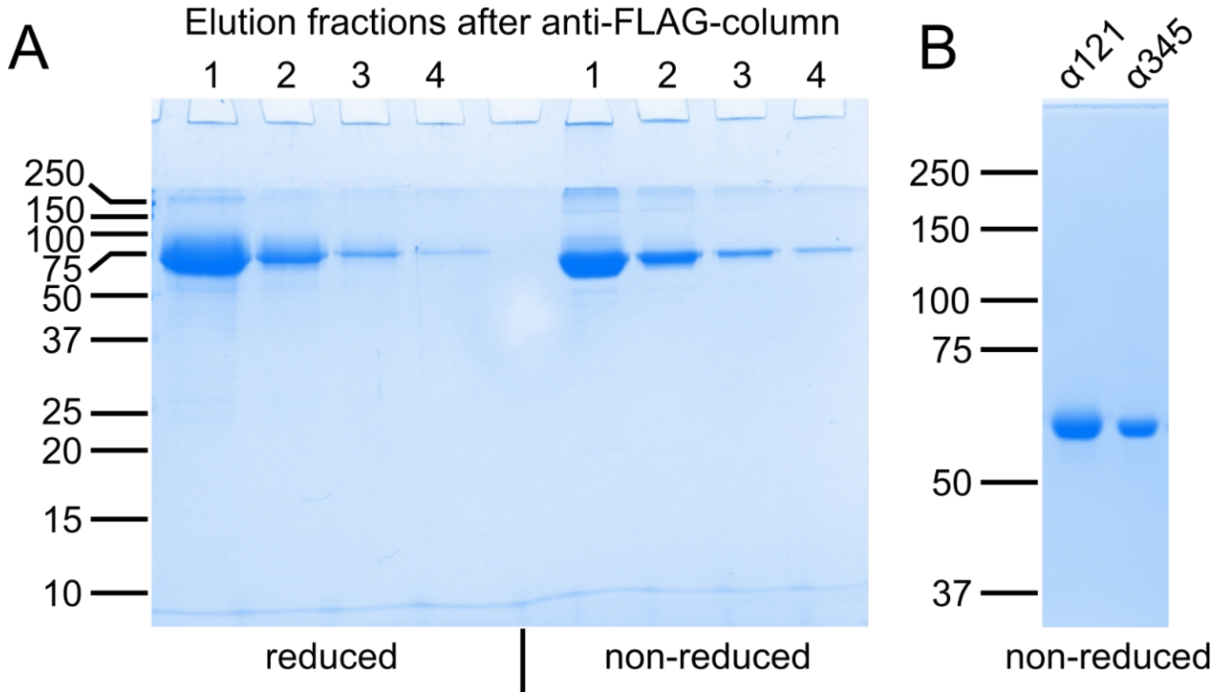

Supplementary Fig. 3. (A) Purification of the single-chain α345 NC1 trimer on anti-FLAG-column showing serial elution fractions with FLAG peptide. Molecular mass of non-reduced α345 NC1 corresponds to a trimer, demonstrating that all disulfides are formed within the trimer. (B) Purified samples of single-chain α121 and α345 NC1 trimers after size-exclusion chromatography demonstrate consistent molecular masses under non-reducing condition.

Supplementary Table 1. Data collection and refinement statistics

**Data collection statistics**	
Wavelength (Å)	0.9786
Resolution range (Å)[a]	68.60-1.76 (1.79-1.76)
Space group	$P4_1\ 2_1\ 2$
Unit cell dimensions (Å)	a=b=128.4, c=104.71
Unit cell angles (°)	α=β=γ=90
Total reflections	711,994 (36,982)
Unique reflections	86,926 (4,534)
Redundancy	8.2 (8.2)
Completeness (%)	100.0 (100.0)
R_{meas} (%)	8.9 (52.1)
I/σI	13.1 (3.4)
**Refinement statistics**	
Resolution range (Å)[a]	45.40-1.76 (1.79-1.76)
Unique reflections used in refinement[b]	86,814 (5,993[c])
R_{work} (%)	13.9 (19.7)
R_{free} (%)[d]	16.2 (23.0)
Bond distance (Å)[e]	0.0059
Bond angle (°)[e]	0.8962
Chiral center (Å^3)[e]	0.0570
Planar group (Å)[e]	0.0061
Dihedral angle (°)[e]	18.7408
Number of atoms (excluding hydrogens)	6,429
Protein atoms	5,389
Chloride ions	6
Polyethylene glycol molecules/atoms	87/684
Water molecules	364
Mean B-value (Å^2)	30.5
Mean protein B-value (Å^2)	25.7
Mean chloride ion B-value (Å^2)	19.5
Mean polyethylene glycol molecule B-value (Å^2)	64.7
Mean water B-value (Å^2)	36.5
**Ramachandran statistics**	
Favored (%)	97.5
Additionally allowed (%)	2.5
Outliers (%)	0

[a] Data for highest resolution shell are given in parentheses
[b] Data cutoff σF=1.34
[c] In working set
[d] 2.3% (2,000 reflections) of data excluded from refinement and used for R_{free}
[e] Root mean square deviation

Supplementary Table 2. Structural comparison of α chains of the α345 crystal structure (present report, PDB ID 6wku) with the corresponding chains in the α121 structure (PDB ID 6mpx). Superimpositions were performed for C_α atoms. *-first α1 domain; #-last α1 domain

α chains	α345 vs α121	α3 vs α1*	α4 vs α2	α5 vs α1#
r.m.s.d., Å	0.67	0.55	2.03	0.54

Supplementary Table 3. Structural comparison of NC1 α chains in homo-oligomeric complexes(1) with the corresponding structures in the α345 hexamer (present report, PDB ID 6wku). Superimposition were performed for C_α atoms.

α chain (PDB id)	α3 hexamer (5nb0)								α4 octamer (5nb1)						α5 hexamer (5naz)
chain ID	A	B	C	D	E	F	G	H	A	B	C	D	E	F	A
r.m.s.d., Å	0.58	0.60	0.59	0.58	0.57	0.57	0.58	0.55	4.73	4.71	4.50	4.66	4.71	4.74	0.58

Supplementary Section 3: The chloride ions at the NC1 α345 trimer-trimer interface

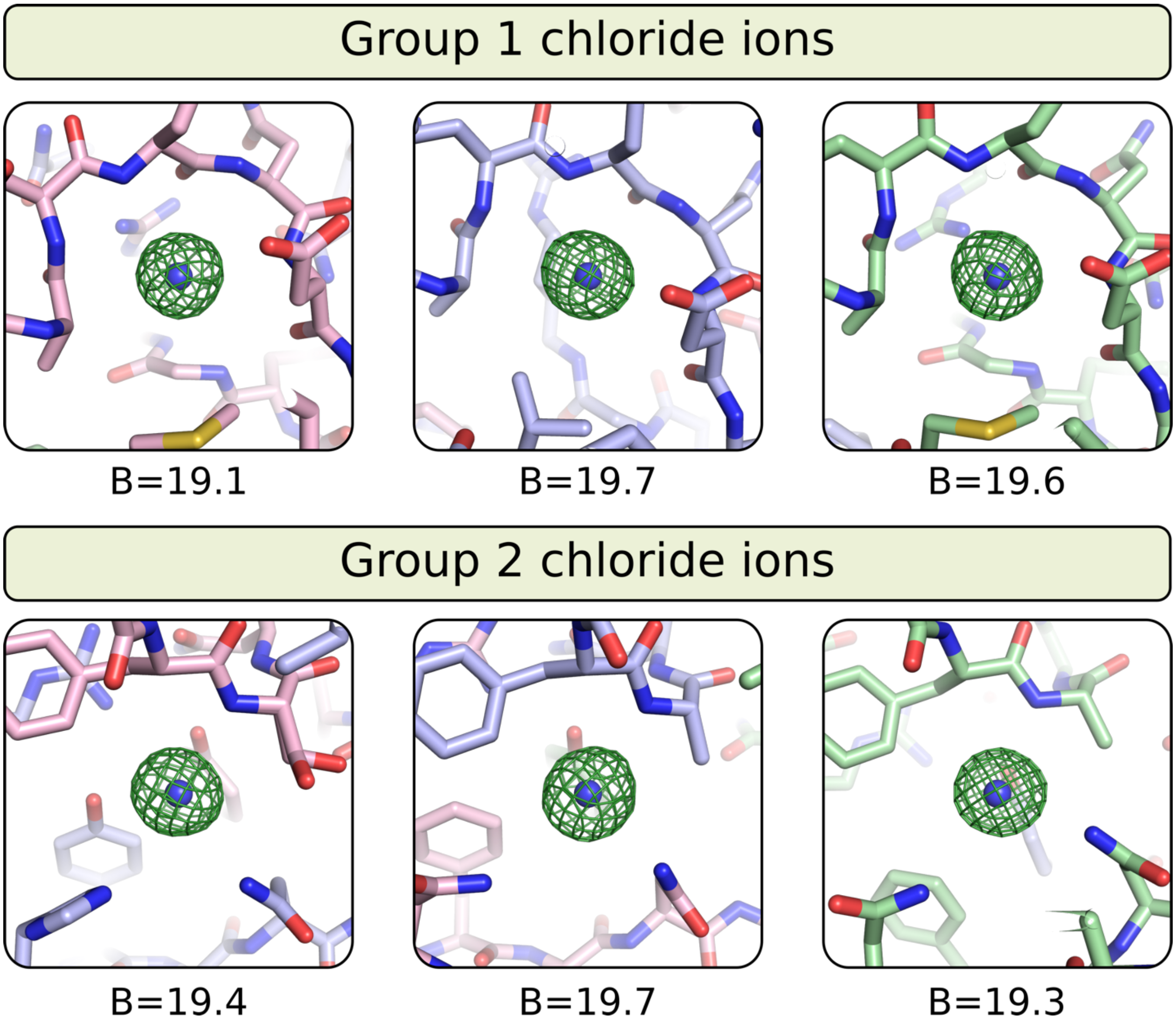

Supplementary Figure 4. *Electron density map of chloride ions*. All six Cl^- ions (twelve per hexamer) have comparable densities and atom displacement values (B values, $Å^2$). Ions are shown as **blue spheres**. The **dark green** meshwork represents the F_o-F_c electron density map calculated before fitting chloride ions (contoured at σ=3). Carbon atom coloring in NC1 chains is **light red** for α3, **light blue** for α4, and **light green** for α5.

Supplementary Table 4. Coordination distances for two groups of chloride ions. Shown are distances between Cl^- ions and atoms involved in hydrogen bonding or ionic interactions (**bold**). Atoms that belong to the opposite trimer are marked with *. Water molecules coordinated by the opposite trimer are also marked with *, whereas water and polyethylene glycol molecules coordinated by both trimers are marked with $^{\#}$.

Group 1

Cl^- #1		Cl^- #2		Cl^- #3	
A74, Cα	3.7 Å	A298, Cα	3.9 Å	A523, Cα	3.6 Å
R76, N	3.4 Å	R300, N	3.2 Å	R525, N	3.5 Å
D78, N	3.3 Å	D302, N	3.4 Å	D527, N	3.3 Å
G178, Cα	3.7 Å	L284, Cδ2	3.7 Å	G627, Cα	3.7 Å
**R179, Nη2**	**3.6 Å**	R401, Cγ	3.8 Å	***R628, Nη1**	**3.4 Å**
		R401, Cδ	3.7 Å		
H_2O, O	3.1 Å	H_2O, O	3.0 Å	H_2O, O	3.0 Å
$^{\#}H_2O$, O	3.4 Å	H_2O, O	3.1 Å		

Group 2

Cl^- #4		Cl^- #5		Cl^- #6	
Y185, Cδ1	4.0 Å	F408, Cδ1	3.6 Å	Y634, Cδ1	3.8 Å
Y185, Cβ	4.0 Å	F408, Cβ	3.9 Å	Y634, Cα	3.8 Å
S186, N	3.2 Å	A409, N	3.2 Å	A635, N	3.2 Å
S186, Cβ	3.5 Å	A409, Cβ	3.6 Å	A635, Cβ	3.8 Å
*Y189, Oη	3.2 Å	*Y638, Oη	3.1 Å	*Y412, Oη	3.2 Å
*Y288, Oη	3.1 Å	*F64, Cε	3.8 Å	*F513, Cε	3.9 Å
*N290, Nδ2	3.4 Å	*N66, Nδ2	3.2 Å	*N515, Nδ2	3.3 Å
		*N66, Cβ	3.9 Å		
$^{\#}$PG4, C2	3.5 Å	$^{\#}$PGE, C1	3.6 Å	$^{\#}$PGE, C5	3.8 Å
$^{\#}$PG4, C1	3.7 Å	$^{\#}$PGE, C2	3.8 Å	$^{\#}$PGE, C6	4.0 Å
		*H_2O, O	3.3 Å	*H_2O, O	3.3 Å
				$^{\#}H_2O$, O	3.4 Å

Supplementary Section 4: Crevices, pockets, inner cavities, loops and surface-exposed reactive residues in the α345 hexamer

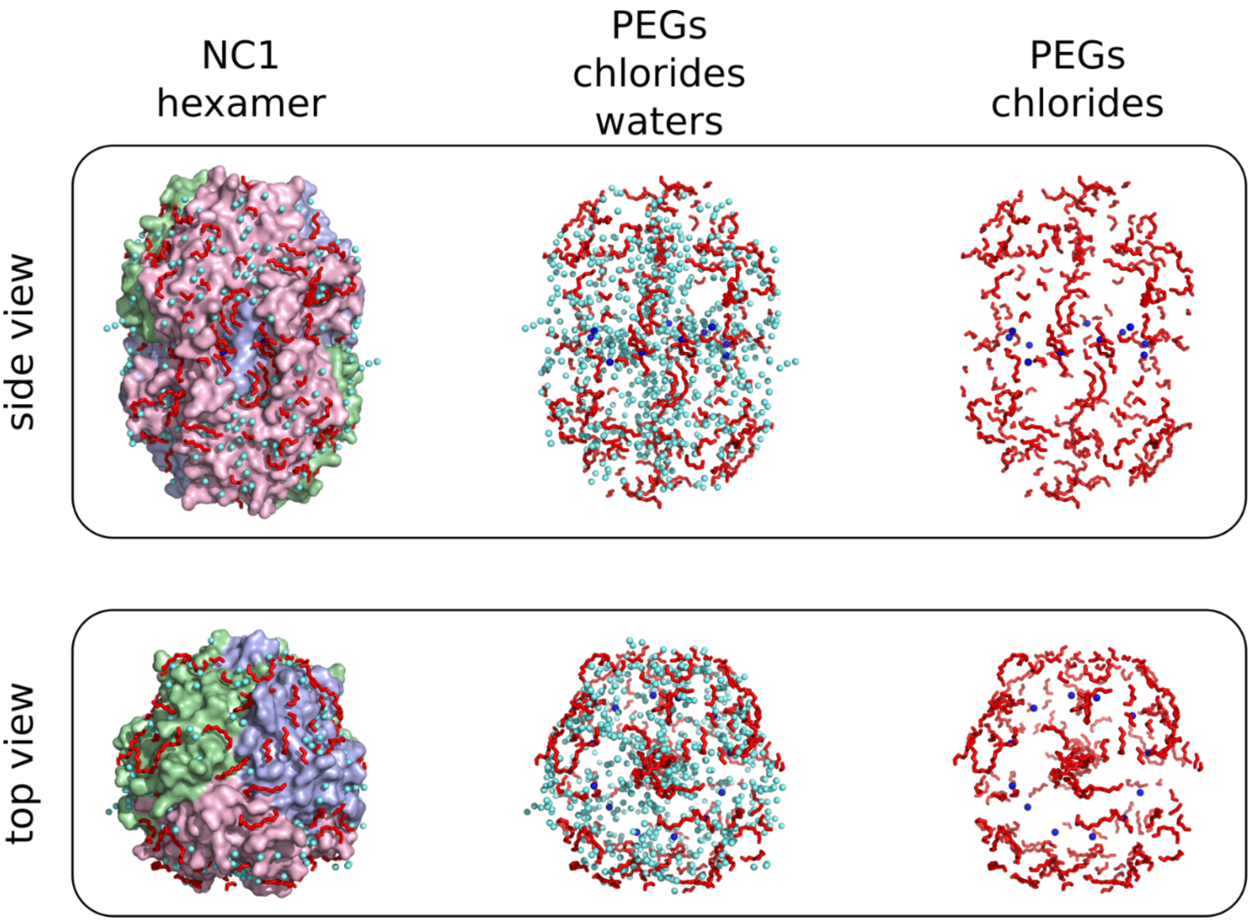

Supplementary Fig. 5. *Structured polyethylene glycol and water molecules on the surface of the α345 hexamer.* Coloring of NC1 chains is **light red** for α3, **light blue** for α4, and **light green** for α5. Chloride ions of both group 1 and group 2 are shown as **blue** spheres. Polyethylene glycol molecules are shown as **red** wireframes. Water molecules are shown as **cyan** spheres.

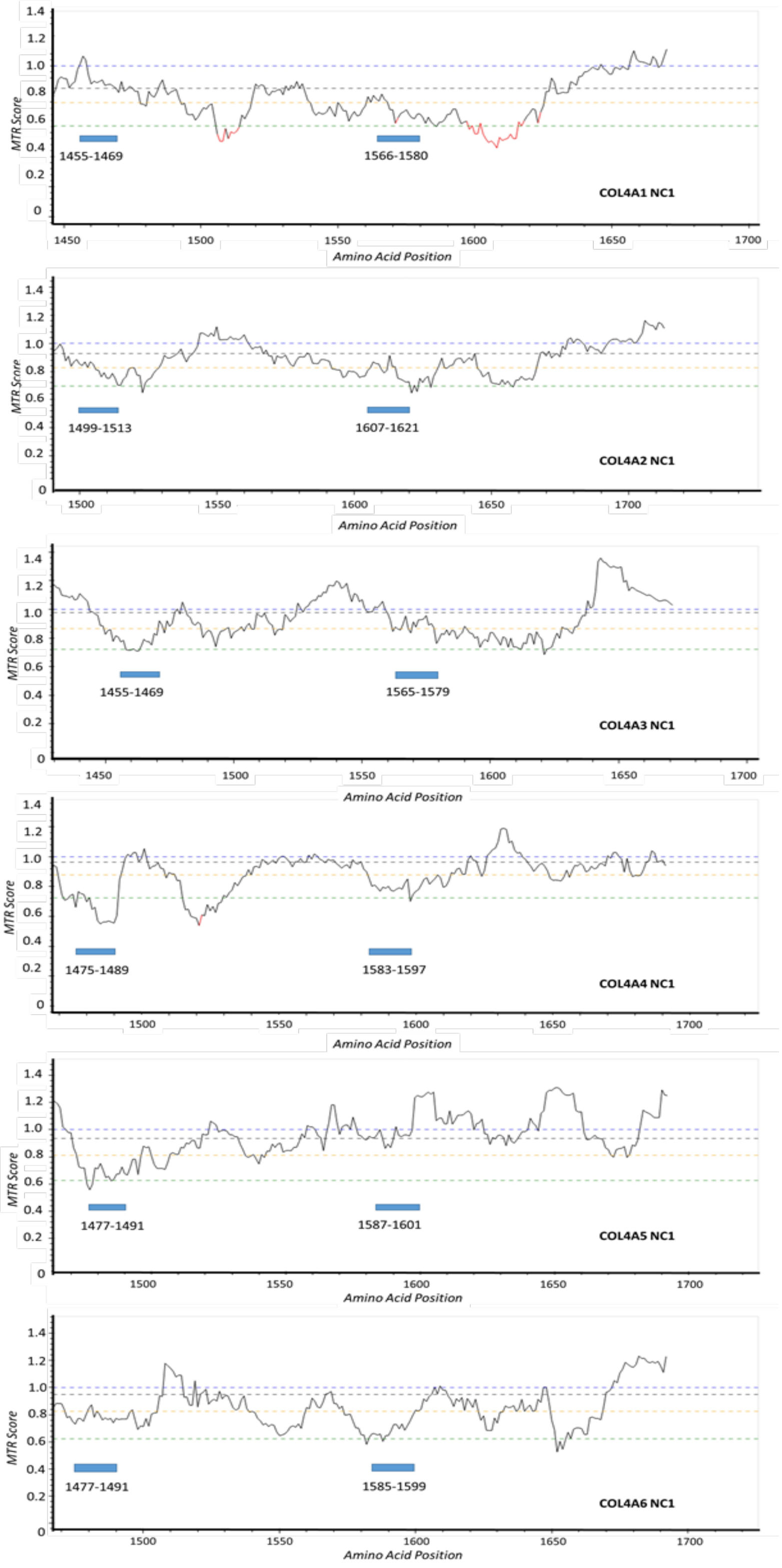

Supplementary Fig. 6. *Missense Tolerance Ratio (MTR) plots for NC1 domains of α1-6 chains of collagen IV generated with MTR-Viewer (21-codon window)(2).* Horizontal dotted lines correspond, from top to bottom, to MTR=1 (no selective pressure) and gene-specific 50th, 25th and 5th MTR percentiles. Amino acid positions for E_A (left) and E_B (right) loops are indicated by blue boxes.

Supplementary Section 5. Analysis of known Alport variants in COL4A3, COL4A4, and COL4A5.

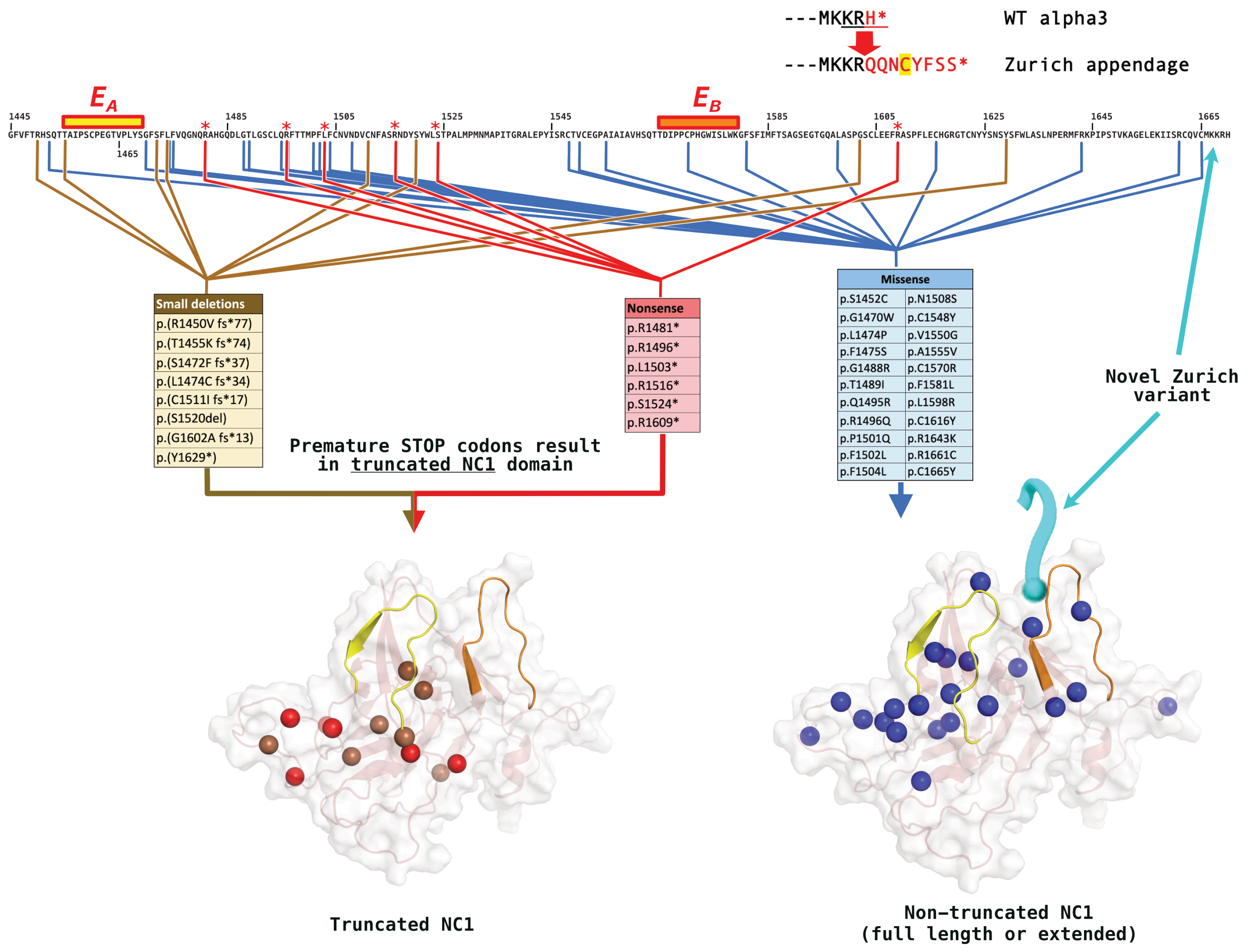

Supplementary Fig. 7. *Crystal structure of α345 hexamer reveals the location of known Alport variants in the α3 NC1 domain.* Distinct pathogenic Alport variants were mapped to the α3 NC1 domain sequence encoded by the COL4A3 gene (**top**) of collagen IV and are mapped within the 3D structure (**bottom**) (source: HGMD 2020.1). Missense variants (blue) account for 2/3 (61%, or 22 out of 36) of all variants within NC1 domain (see Supplementary Tables 5 and 6 for details). Small deletions (brown) and nonsense (red) variants result in the premature stop codon and production of the truncated form of COL4A3 (bottom left). While missense variants do not affect overall length of NC1 domain, they may result in conformational changes of crucial regions within NC1. According to the HGVS nomenclature, novel Zurich variant (cyan) belongs to a "small deletion" subtype of variants. But in contrast to the other known deleterious mutations, Zurich variant adds an 8-aa appendage, extending the NC1 domain, and thus belongs to "Non-truncated" subgroup.

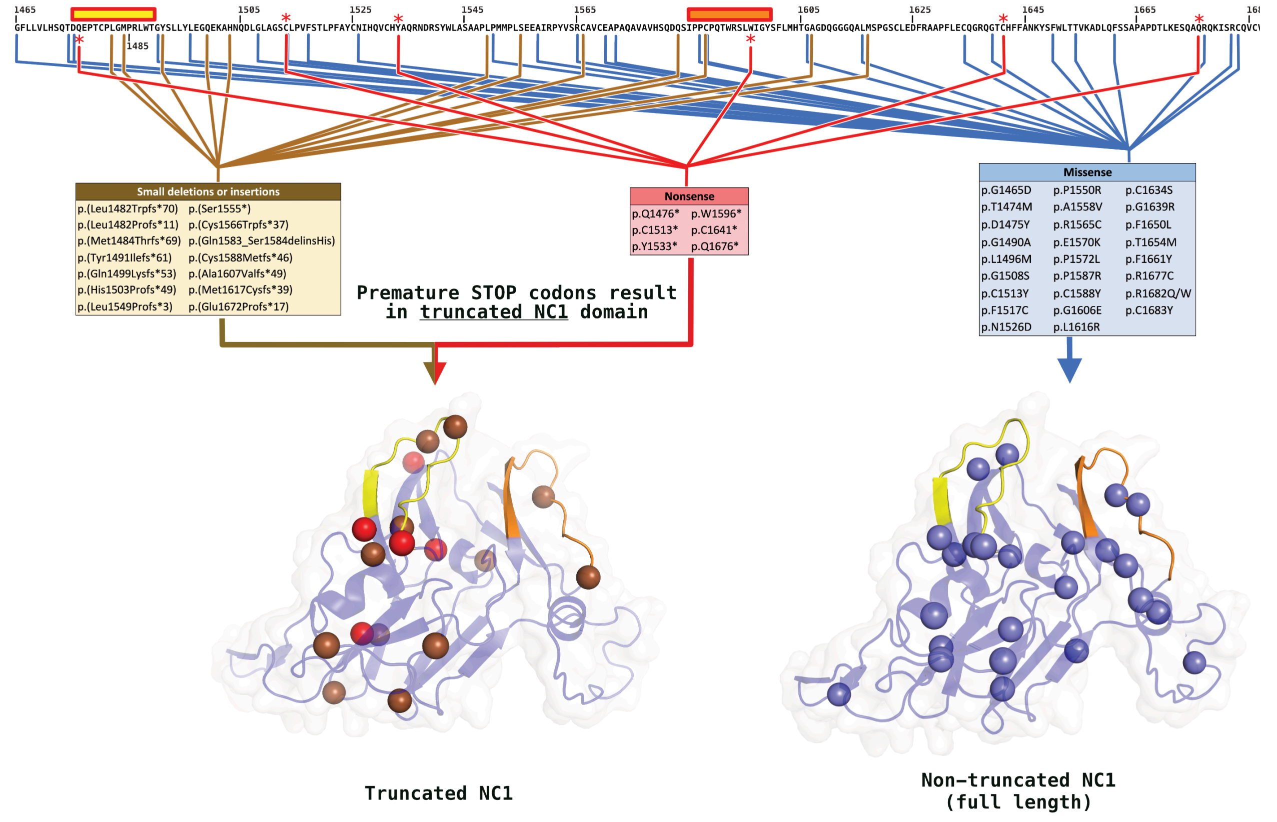

Supplementary Fig. 8. *Crystal structure of α345 hexamer reveals the location of known Alport variants in the α4 NC1 domain.* Distinct pathogenic Alport variants were mapped to the α4 NC1 domain sequence encoded by the COL4A4 gene (**top**) of collagen IV and are mapped within the 3D structure (**bottom**) (source: HGMD 2020.1). For detailed description and color codes see the legend to Supplementary Figure 7.

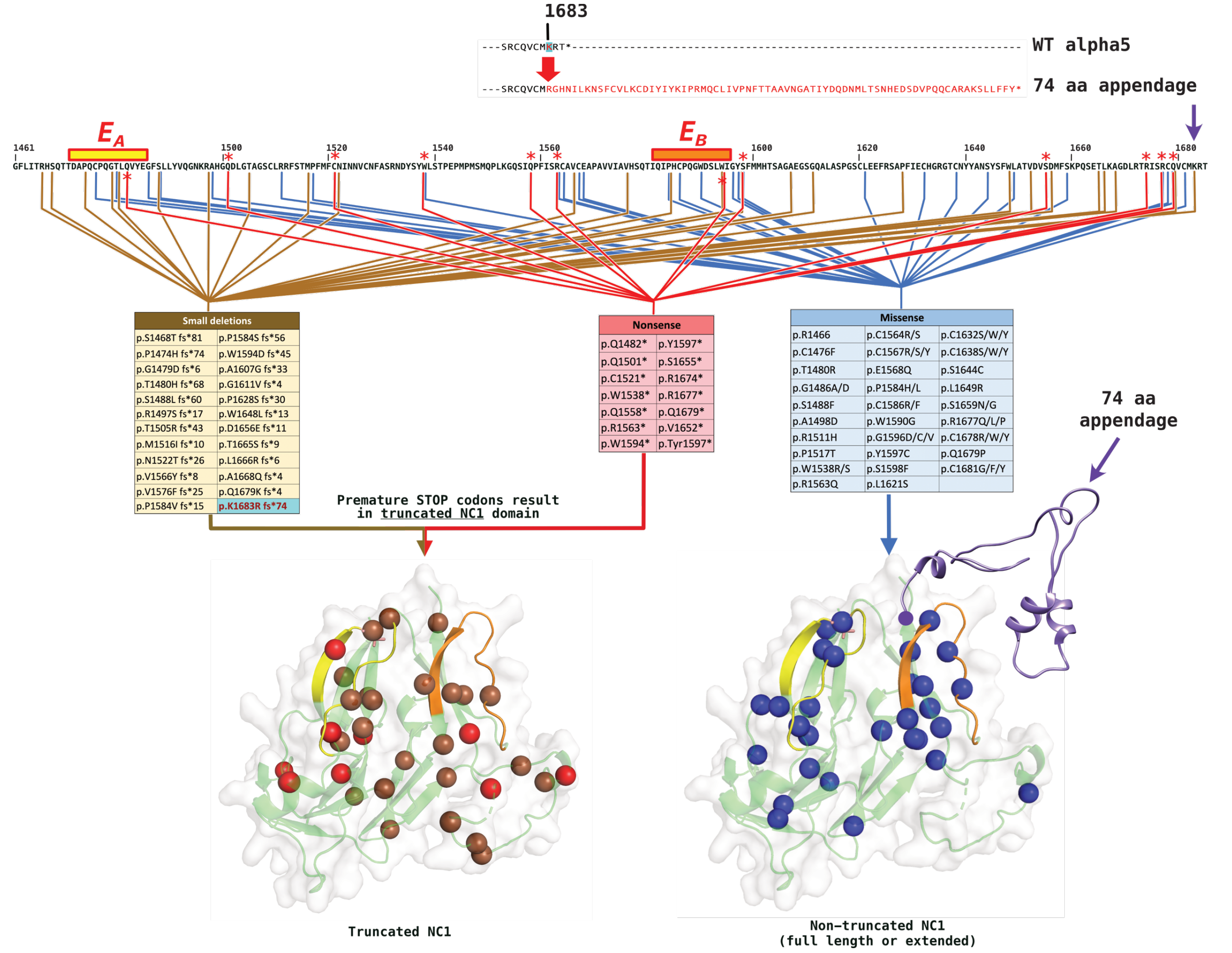

Supplementary Fig. 9 *Crystal structure of α345 hexamer reveals the location of known Alport variants in the α5 NC1 domain.* Distinct pathogenic Alport variants were mapped to the α5 NC1 domain sequence encoded by the COL4A5 gene (**top**) of collagen IV and are mapped within the 3D structure (**bottom**) (source: HGMD 2020.1). A 74-amino acid appendage at the C-terminus of α5 chain (residue K^{1683}) is depicted in the middle. For detailed description and color codes see the legend to Supplementary Figure 7.

Supplementary Table 5. Pathogenic variants of various types in the coding regions of COL4A3, COL4A4, and COL4A5 genes. Source: HGMD 2020.1

HGMD 2020.1

Mutation type	COL4A3	COL4A4	COL4A5	TOTAL
Missense/nonsense	180	164	557	901
Splicing substitutions	41	30	206	277
Regulatory substitutions	0	1	0	1
Small deletions	41	43	179	263
Small insertions/duplications	14	12	65	91
Small indels	2	2	11	15
Gross deletions	11	8	139	158
Gross insertions/duplications	3	2	6	11
Complex rearrangements	0	0	8	8
Repeat variations	0	0	0	0
**TOTAL**	**292**	**262**	**1171**	**1725**

Supplementary Table 6. Pathogenic variants in NC1 domains of COL4A3, COL4A4, and COL4A5. Source: HGMD 2020.1. Missense variants do not change overall length of the NC1 domains.

**NC1 Domains**			
**Variant type**	**COL4A3**	**COL4A4**	**COL4A5**
Missense/nonsense	22/6	29/6	49/14
Small deletions	8	11	18
Small insertions/duplications	-	3	7
Small indels	-	-	1
Terminal codon deletion *(Zurich- and Zurich-like variants)*	1	-	1
**TOTAL**	**37**	**49**	**90**

Supplementary References

1. Casino, P., Gozalbo-Rovira, R., Rodriguez-Diaz, J., Banerjee, S., Boutaud, A., Rubio, V., Hudson, B. G., Saus, J., Cervera, J., and Marina, A. (2018) Structures of collagen IV globular domains: insight into associated pathologies, folding and network assembly. *IUCrJ* **5**, 765-779
2. Silk, M., Petrovski, S., and Ascher, D. B. (2019) MTR-Viewer: identifying regions within genes under purifying selection. *Nucleic Acids Res* **47**, W121-W126
